# Structural network topology and cognitive control in very preterm born young adults

**DOI:** 10.1016/j.nicl.2026.103952

**Published:** 2026-01-20

**Authors:** Merle J. Marek, Dieter Wolke, Christian Sorg, Jil Wendt, Aurore Menegaux, Dennis Hedderich, Peter Bartmann, Micha Burkhardt, Andrea Hildebrandt, Axel Heep

**Affiliations:** a Psychological Methods and Statistics, Department of Psychology, Carl von Ossietzky Universität Oldenburg, Germany; b Department of Psychology, University of Warwick, Coventry, UK; c Warwick Medical School, University of Warwick, Coventry, UK; d Institute for Neuroradiology, TUM University Hospital, Technical University of Munich, School of Medicine and Health, Munich, Germany; e TUM-NIC Neuroimaging Center, Technical University of Munich, School of Medicine and Health, Munich, Germany; f Department of Psychiatry, School of Medicine and Health, Technical University of Munich, Munich, Germany; g Department of Neonatology and Paediatric Intensive Care, University Hospital Bonn, Bonn, Germany; h Research Centre Neurosensory Science, Carl von Ossietzky Universität Oldenburg, Germany; i Paediatrics, Department of Human Medicine, School of Medicine and Health Sciences, Carl von Ossietzky Universität Oldenburg, Germany

**Keywords:** Very preterm birth, Cognitive control, Graph theory, Structural connectome, Integration and segregation, Structural equation modelling

## Abstract

This study investigates the long-term effects of very preterm (VPT) birth (<32 weeks of gestation) on cognitive control and structural brain network topology. Data were obtained from 61 very VPT and 79 full term (FT) individuals aged 26 years, who participated in the prospective Bavarian Longitudinal Study since birth (BLS, https://www.bayerische-entwicklungsstudie.de). Cognitive control ability was estimated through a multi-group confirmatory factor analysis, while the structural topology of the cognitive control brain network was analysed through diffusion-weighted magnetic resonance imaging, tractography, and graph-theoretic analysis. Brain-behaviour associations were further examined using structural equation modelling. Consistent with expectations, we observed significant group differences in cognitive control, especially in a latent *speed of cognitive control* factor, with FT individuals scoring 0.742 standard deviations higher than the VPT group (*p* = 0.001). This was statistically confirmed by a model comparison in the multi-group framework. Additionally, the VPT group exhibited lower structural network integration, as indicated by reduced global efficiency and average degree of the cognitive control brain network. Contrary to our hypothesis, we found no group differences in network segregation (average clustering coefficient), and structural network characteristics were not significantly associated with latent cognitive control. These findings indicate cognitive control deficits and reduced structural brain network integration in adulthood for VPT individuals and underscores the need for ongoing support and intervention to mitigate the lasting neurodevelopmental impacts of preterm birth.

## Highlights

• Very preterm birth (VPT) is associated with lower cognitive control in adulthood.
• VPT adults show reduced structural brain network integration & similar segregation.
• Brain–behaviour associations were absent, except in a small subcortical network.

## 1 Introduction

Preterm (PT) birth, defined as birth before 37 weeks of gestation, accounts for approximately 10% of deliveries worldwide (Walani, 2020). PT born infants often experience challenges in attention regulation, cognition, and executive functions (Aarnoudse-Moens et al., 2009, Mulder et al., 2009, Réveillon et al., 2018) in later life. These differences remain even among those born very PT (VPT; <32 weeks GA) without severe disabilities (Breeman et al., 2016). Despite advancement in neonatal care, extremely PT (<28 weeks GA) children have not shown improvements in executive functions over the past three decades (Burnett et al., 2018, Burstein et al., 2024). Less is understood about the potential long-term impacts of PT birth on the brain into adulthood. Impairments in cognitive control have been reported in adolescents (Burnett et al., 2018) and adults (Breeman et al., 2016, Eryigit-Madzwamuse et al., 2015b, Fernández de Gamarra-Oca et al., 2023). These impairments pose significant risks, as cognitive control issues are prevalent in psychiatric and neurological conditions such as attention deficit hyperactivity disorder (ADHD; e.g., Boonstra et al., 2005) and depression (Snyder, 2013), and are closely linked to academic performance (Best et al., 2011). Our study aims to examine the long-term association between gestational age (GA) at birth and cognitive control and specifically the neural correlates underlying any observed associations.

Cognitive control refers to the flexible allocation of attentional resources and other low-level cognitive processes during goal-directed behaviour (Inzlicht et al., 2021, Miyake et al., 2000). Although often used interchangeably with executive functions, cognitive control can be understood more broadly. It encompasses basic executive functions — such as response inhibition, working memory updating, and mental set shifting — as well as more complex functions like planning and emotion regulation (Crone and Steinbeis, 2017). Withholding inappropriate responses to internal or external distractions while maintaining focus on a predefined goal is fundamental, and arguably the most critical component of cognitive control (Bastian et al., 2020). Response inhibition has even been proposed as a core facet of cognitive control due to its shared variance with other basic cognitive control domains like working memory updating and mental set shifting (Friedman and Robbins, 2022). Thus, conflict monitoring and attention are central aspects of cognitive control (Bastian et al. 2020, Botvinick et al., 2001).

Cognitive control has traditionally been explained in terms of underlying neural activity within certain networks, such as the multiple-demand system, due to its domain-general role in cognitive control tasks (Fedorenko et al., 2013, Langner and Eickhoff, 2013, Langner et al., 2018, Niendam et al., 2012). This view builds upon the notion that complex processes like cognitive control rely on a broad network of brain regions to support flexible information flow (Tang and Bassett, 2018). The cognitive control or multiple-demand network includes the dorsolateral prefrontal cortex (PFC), anterior and mid-cingulate cortex (ACC/MCC), insula, precentral gyrus, (pre-) supplementary motor area, intraparietal sulcus, and other parietal regions, as well as a small occipital region and the cerebellum. Subcortically, the network encompasses the thalamus, putamen, and caudate nucleus.

Understanding the role of large-scale brain networks implicated in complex cognitive processes and behaviour requires a focus on the interplay between different network regions rather than solely their activation. Anatomical networks have been described as ‘the skeleton that constrains the passage of neuronal signalling and information that is crucial for shaping our thoughts, understanding, and actions’ (Petersen and Sporns, 2015). Potential communication pathways within a network can be mathematically described using graph theory, representing the brain as a network of nodes connected by edges (Rubinov et al., 2010). In structural connectome analyses, nodes are defined as brain areas defined through parcellation of the brain, also called regions of interest (ROIs). Edges represent white matter fibre tracts between these ROIs, e.g. traced via tractography algorithms. Measures that describe the relationships between the nodes quantify the topology of these networks and thus provide insights into how the brain’s architecture may support the communication of information. These measures can be broadly categorised either as measures of segregation, which capture the efficiency of local information flow, or as measures of integration, reflecting the efficiency of information transfer across the network (Bullmore and Bassett, 2011). Additionally, the centrality of individual nodes can be assessed to determine their local importance within the network, offering insights into their role in facilitating information processing.

PT birth has immediate impacts on the development of structural brain networks (Inder et al., 2023, Quinones et al., 2023) and their topology (Ball et al., 2014, Batalle et al., 2017). PT neonates (de Almeida et al., 2021) and school children (de Kieviet et al., 2012) demonstrate higher segregation and lower integration in structural networks, particularly within fronto-limbic connections of the cognitive control circuit. Differences in network topology and node centrality within striatal and frontal regions of the cognitive control circuit appear to persist into late adolescence (Irzan et al., 2021). While the long-term effects of PT birth on the brain are less well understood, certain structural alterations — such as volume reductions in thalamic structures and the basal ganglia —have been found to continue into adulthood (Kelly et al., 2023). Additionally, cognitive performance has been associated with microstructural properties of white matter tracts in PT-born adults (Allin et al., 2011, Menegaux et al., 2020, Meng et al., 2016). In summary, PT birth appears to have enduring consequences on the brain. Here, we investigate whether these effects also shape the topology of the structural cognitive control network.

In our study, we utilised a dataset of VPT-born young adults from the Bavarian Longitudinal Study (BLS, https://www.bayerische-entwicklungsstudie.de) to investigate the long-term consequences of VPT birth on cognitive control. First, we examined whether there are differences in cognitive control behaviour and in the topology of the structural cognitive control brain network in adulthood between VPT- and full term (FT)-born individuals. We hypothesised that VPT-born young adults exhibit lower levels of cognitive control compared to their FT-born peers (Hypothesis 1). Secondly, in line with previous findings on PT-born children, we hypothesised that the structural cognitive control brain network of VPT-born young adults shows greater segregation and lower integration (Hypothesis 2) compared to that of the FT-born group. Due to limited power, group differences in node centrality were explored in a preregistered exploratory analysis. Lastly, we investigated whether network topology predicts cognitive control. Accordingly, we hypothesised that the topology of the cognitive control brain network is associated with cognitive control on the behavioural level in both VPT- and FT-born young adults (Hypothesis 3). These hypotheses and the methodology were preregistered (see: osf.io/k4xwa).

## 2 Methods

### 2.1 Participants

We used a subset of the dataset from the BLS. The BLS is a prospective, longitudinal study that has been following individuals born VPT or with very low birth weight as well as healthy term born infants in southern Bavaria from birth to adulthood since 1985. A detailed description of the study design was published in Wolke et al. (1994). Data was collected at nine time points across six developmental stages (neonates, 5 months (m), 20 m, 56 m, 6 years (y), 8 y, 12/13 y, 25/26 y, 34 y, and 38 y). Participants were categorised based on GA and birth weight. The BLS target sample included all infants born alive and at risk in southern Bavaria, Germany, between February 1, 1985, and March 31, 1986. The VPT group inclusion criteria were a birth weight < 1500 g or GA < 32 weeks, with admission to one of 16 regional children’s hospitals within 10 days after birth (*N_VPT_* = 682). Healthy, FT newborns, were recruited from postnatal wards in obstetric hospitals in the same area during the same time frame as the VPT sample. The FT group was selected through stratified random sampling (*N_FT_* = 916) to match the socio-economic profile of the VPT sample. For a comprehensive description of the study cohort, see Wolke et al. (2009).

In our analyses, we considered only data collected from participants at age 26 who had survived and were living in Germany. In addition to behavioural and survey data, magnetic resonance imaging (MRI) data were collected at this time point. This led to additional exclusion criteria, including claustrophobia, inability to remain still for over 30 min, certain medical conditions, pregnancy, non-removable metal implants, and severe neurological trauma (Menegaux et al., 2021). At this time point, 217 VPT and 197 FT participants completed the behavioural and cognitive assessments, of whom 73 VPT and 89 FT participants also underwent MRI scanning, including diffusion imaging. Eight VPT and eight FT participants were excluded due to unsatisfactory MRI data quality prior to this analysis.

Ethical approval for the study was granted by the University of Munich Children’s Hospital, Germany. The ethics committees of Klinikum rechts der Isar and the University Hospital Bonn approved the inclusion of MRI-related parameters. MRI data were acquired at two study sites: the Department of Neuroradiology at Klinikum rechts der Isar and the Department of Radiology at University Hospital Bonn. Participants provided informed consent and received compensation for travel expenses and attendance.

### 2.2 Behavioural and demographic data

#### 2.2.1 Indicators of cognitive control

Cognitive control ability was estimated as a latent factor indicated by various behavioural tasks, expert observations, and questionnaires, which will be briefly explained below. The tasks include the German version of the Colour-Word Interference or Stroop test (Eryigit-Madzwamuse et al., 2015a, Bäumler, G., 1985), which comprises three tasks measuring verbal inhibition, cognitive flexibility, and selective attention. In the first task, participants were asked to read the colour of a presented word printed in black (‘word’). In the second task, participants were instructed to name the colour of presented colour bars (‘bar’). Thirdly, participants were asked to name the colour of presented colour words, irrespectively of the word that is printed, which could be either congruent or incongruent (‘interference’). The primary measurements were the time taken by participants to complete each task. Each of these time/speed of cognitive control variables was normatively transformed to *T*-scores (see Bäumler, G., 1985).

The Attention Network Task (ANT; Eves et al., 2020, Posner et al., 1990) was originally designed to test three neural attentional networks in children and adults. One of these networks is the executive control or congruence network, which relates to the ability to resolve conflicts among responses. Participants respond by pressing two keys indicating the direction (left or right) of a central arrow surrounded by congruent or incongruent flankers. The network’s efficiency is represented by a congruence score (‘congruency’), which is calculated as the differences in response time between congruent and incongruent conditions, where a higher score in milliseconds indicates lower efficiency. To align with other indicators where higher values reflect better performance, this variable was inverse scaled. In addition to the executive control network, the ANT task measures an alerting and an orienting network. However, variables linked to these networks were excluded from the latent cognitive control ability estimation in this analysis due to their weak, non-significant, and negative factor loadings, as well as their limited theoretical relevance to cognitive control in this context.

In the Visual Search and Attention Test (VSAT; Eryigit-Madzwamuse et al., 2015a, Trenerry et al., 1990), participants were presented with four arrays of letters. In each trial, they were instructed to quickly scan the array and cross out all items matching a specified target within a 60-second time limit. An overall attention score, based on the total number of correct cancellations for two trials, was standardised against group norms computed from control participants within the BLS and subsequently normalised (‘vsat’).

As described in Breeman et al. (2015), participants’ attention span during cognitive tasks throughout the assessment day was rated by psychologists using the Tester’s Rating of Adult Behavior (TRAB; Wolke, D [Dieter], 2012). Scores range from 1 (very short attention span) to 9 (very long attention span). Attention span was rated three times over the day, and the final score is the average of these ratings (‘trab’). In the measurement model of cognitive control ability, the TRAB score is treated as a quantitative variable.

Parents were also asked to rate their children’s attention problems at age 26 using the Young Adult Behavior Checklist (YABCL; Achenbach, 1997). This checklist includes items describing typical behaviours, with parents indicating the frequency of each behaviour on a scale from 0 (not true) to 2 (very/often true). Ratings across items were summed to generate a total score indicative of general attention problems. To align with other cognitive control indicators, YABCL scores were rescaled so that higher scores reflect better performance (‘yabcl’).

Eryigit-Madzwamuse et al. (2015a) and Breeman et al. (2016) used data from the BLS young adult sample to compare Stroop and VSAT performance as indicators of executive function, as well as the YABCL and TRAB. They reported group differences with moderate to large effect sizes. In the present study a subset of this sample was analysed; however, with differences from the previous studies in two fundamental ways. First, this study examined a latent variable representing general cognitive control that encompasses multiple indicators and captures shared variance across them. Second, with a focus on brain-behaviour associations, our study specifically includes only participants who also met MRI testing criteria, creating a more specific subsample due to additional exclusions for excessive movement. We expect that group differences will be confirmed in this refined subset of VPT and FT adults.

#### 2.2.2 Covariates

Motor impairments were assessed at 6 and 8 years using the Revised Test of Motor Impairment (TOMI, Stott et al., 1984). This instrument evaluates motor deficits across 10 items, with scores for individual items aggregated into a total sum score. The total score was subsequently transformed into a TOMI sum score. This transformation provides an indicator of the extent to which a motor-impaired child deviates below age-matched peers, based on normative data derived from the BLS sample.

Perinatal data, including sex, GA at birth (in weeks) and birth weight, were extracted from maternity clinic perinatal records. GA was estimated based on maternal reports of the last menstrual period and serial ultrasounds conducted during pregnancy. If the two measures differed by more than two weeks, additional clinical assessment using the Dubowitz method (Dubowitz et al., 1970) was performed after birth. Additionally, family socioeconomic status (SES) was included as a covariate, given its moderating influence on the relationship between GA and cognitive outcomes (Fernández de Gamarra-Oca et al., 2023, Mulder et al., 2009). Following previous studies using this dataset (Breeman et al., 2015, Eryigit-Madzwamuse et al., 2015a), SES was indexed as a weighted composite score derived from parental education and occupation. Four additional covariates were included to account for potential influences on outcomes: head circumference at birth (in cm), postnatal infant respiratory distress (ventilation disorder), and the INTI (intensity of neonatal treatment) index based on daily clinical ratings by research nurses in the neonatal intensive care unit. The INTI is calculated as the mean score from daily assessments of care level, respiratory support, feeding dependency, and neurological status (scored on a 0–3 scale). Notably, INTI scores and ventilation disorder are only present in the VPT group. Finally, the summed perinatal optimality score was derived from the optimality questionnaire designed to capture the overall perinatal burden experienced by the mother and the newborn.

### 2.3 Neuroimaging data

#### 2.3.1 Acquisition and harmonisation

The acquisition of MRI data was previously described by Meng et al. (2016). For the present analysis, structural T1-weighted (T1w) images and diffusion-weighted imaging (DWI) data were utilised, as DWIs measure water molecule diffusion, allowing for the reconstruction of structural brain networks and connectivity patterns. Some T1w and DWI data were acquired on 3 T Philips Achieva scanners with standard 8-channel SENSE head coils in Munich and Bonn and some data were acquired on 3 T Philips Ingenia systems (see Table 1 for frequencies per scanner). As per Meng et al. (2016), T1w images were acquired with a magnetization-prepared rapid acquisition gradient echo (MPRAGE) sequence. Sequence parameters were as follows: echo time (TE) = 3.9 ms, repetition time (TR) = 7.7 ms, flip angle = 15°, field of view = 256 x 256 mm^2^, matrix = 256 x 256, 180 sagittal slices, slice thickness = 1 mm, and 0 mm interslice gap, voxel size = 1 x 1 x 1 mm^3^. DWIs were acquired with a single-shot spin-echo echo-planar imaging sequence with TE = 47 ms, TR = 20,150 ms, flip angle = 90°, field of view = 224 x 224 mm^2^, matrix = 112 x 112, 75 transverse slices, slice thickness = 2 mm, and 0 mm interslice gap, voxel size = 2 x 2 x 2 mm^3^, resulting in one b = 0 s/mm^2^ frame and 32 DWIs with b = 1000 s/mm^2^.

**Table 1:** Demographic data of the participants.

		**FT**	**VPT**	**Total**
		**(*N* = 79)**	**(*N* = 61)**	**(*N* = 140)**
**Assigned sex at birth**	Female	29 (37%)	25 (41%)	54 (39%)
	Male	50 (63%)	36 (59%)	86 (61%)
**GA (weeks)**	mean ± *SD*	40 ± 1	30 ± 1.9	36 ± 5
**Birth weight (g)**	mean ± *SD*	3376 ± 480	1296 ± 324	2470 ± 1116
**Head circumference (cm) at birth**	mean ± *SD*	35 ± 1.2	28 ± 2.7	32 ± 4.3
**Multiple birth**	singleton	76 (96%)	46 (75%)	122 (87%)
	twin	2 (3%)	9 (15%)	11 (8%)
	triplet	1 (1%)	2 (3%)	3 (2%)
	quadruplet	0 (0%)	3 (5%)	3 (2%)
	sextuplet	0 (0%)	1 (2%)	1 (1%)
**SES at birth**	Upper	26 (33%)	12 (20%)	38 (27%)
	Middle	34 (43%)	32 (52%)	66 (47%)
	Lower	19 (24%)	17 (28%)	36 (26%)
**Perinatal burden: summed optimality score**	mean ± *SD*	2.3 ± 1.5	4.8 ± 1.3	3.4 ± 1.9
**MRI scanner**	Bonn Achieva	9 (11%)	3 (5%)	12 (9%)
	Bonn Ingenia	13 (16%)	26 (43%)	39 (28%)
	Munich Achieva	43 (54%)	29 (48%)	72 (51%)
	Munich Ingenia	14 (18%)	3 (5%)	17 (12%)

*Notes*. FT: full term group, VPT: very preterm group, GA: gestational age at birth, *SD*: standard deviation, SES: socioeconomic status.

Given the use of different scanners, neuroimaging data were harmonised using the *neuroCombat* tool (Fortin et al., 2018, https://github.com/Jfortin1/neuroCombat) at the level of the outcome parameter raw streamline count. *NeuroCombat* aims to remove unwanted variability attributable to scanner-related effects while preserving variation associated with biological variables such as sex and group (VPT/FT). The method assumes that the expected value of the outcome parameter can be modelled as a linear combination of scanner and biological effects, incorporating site-specific scaling factors. *NeuroCombat* applies an empirical Bayes approach to harmonize data from multiple sites or scanners. Compared to harmonisation via linear regression, with or without adjustment for biological covariates, *neuroCombat* has demonstrated superior performance.

Due to a strong group imbalance across scanners in our dataset, a confound was expected between scanner and group effects. To mitigate this, we generated five random subsamples with balanced group frequencies across scanners. The Bonn Achieva and Munich Ingenia scanners were excluded due to the small number of participants in the smaller group (*ns_PT_* = 3). For the remaining scanners (Bonn Ingenia and Munich Achieva), we created balanced samples by randomly downsampling the larger group. Each resulting dataset included *N* = 84 participants (*n_Bonn_Ingenia_* = 26: 13 random VPTs and all 13 FTs; and *n_Munich_Achieva_* = 58: all 29 VPTs and 29 random FTs).

To evaluate scanner effects, we applied *neuroCombat* to the reduced samples and re-ran the neural analyses using the harmonised data. Results are provided in the Supplement S1. In summary, results are similar to those without harmonisation of the data. Please note that a lack of statistical significance in some analyses is likely due to reduced power, given the smaller sample size after harmonization. In the main paper, we will, therefore, focus on the full dataset (non-harmonised), while also reporting results after harmonisation.

#### 2.3.2 Preprocessing, and quality control

Preprocessing of structural and DWI data was conducted prior to this analysis, as previously detailed in Wendt et al. (2024). Critically, the entire sample was re-preprocessed using updated, state-of-the-art neuroimaging pipelines to enhance data quality and comparability with more recent datasets. Notably, we implemented distortion correction using SynB0-DisCo (see below) to reproduce the reverse phase-encoded b0 acquisitions required for modern correction techniques, which were not available with the original BLS data. All together, these updated tools and procedures not only improved preprocessing accuracy but enabled the inclusion of additional cases previously excluded due to quality constraints.

Structural images were preprocessed prior to this analysis using the standard FSL (FMRIB software library, Smith et al., 2004) pipeline, including reorientation, cropping, bias field correction, and brain extraction. The images were then denoised using the MRI Denoising Software with the Adaptive Optimized Nonlocal Means option. To preprocess DWI data, the initial stages of the PreQual pipeline were applied, including MP-PCA denoising, inter-scan normalization, and synthetic b0 generation via SynB0-DisCo to compensate for the absence of reverse phase-encoded images for distortion correction. Susceptibility-induced distortions were corrected using FSL Topup, with the synthetic b0 as an anatomical reference. Brain extraction was then performed with FSL BET, followed by eddy current distortion and motion correction, with the option to incorporate outlier replacement and slice-wise signal dropout imputation using FSL EDDY. Transformation matrices for registration between structural, diffusion and Montreal Neurological Institute (MNI) standard space were calculated with the Advanced normalization tools (ANTs) package.

The complete quality control pipeline is detailed in Wendt et al. (2024). Briefly, images were visually inspected for ghosting and chemical shift artefacts, motion, susceptibility-induced distortions, and excessive noise. After preprocessing, FLS QUAD and SQUAD were used to quantitatively assess motion, contrast-to-noise ratio and the percentage of outliers individually and across groups. Additionally, residuals from the FSL DTIFIT-derived diffusion tensor model were examined to evaluate the impact of artefacts. The corrected data underwent another visual inspection and in case of uncertainty in any of the quality control steps, additional DWI quality control experts were consulted.

### 2.4 Cognitive control network

#### 2.4.1 Nodes

Network nodes were defined as ROIs associated with cognitive control, based on previous meta-analyses (Fedorenko et al., 2013, Langner and Eickhoff, 2013, Langner et al., 2018, Niendam et al., 2012). These nodes include dorsolateral prefrontal areas, the ACC/MCC, the insula, distinct parietal regions, the inferotemporal cortex, a small occipital region, and the cerebellum. Subcortically, the cognitive control network includes the thalamus and basal ganglia (putamen and caudate). In total, each hemisphere contained 33 nodes, resulting in 66 nodes per participant. For a detailed list of the ROIs representing the nodes, please refer to Table S2 in the Supplement.

Cortical parcellation was conducted based on the Destrieux atlas (Destrieux et al., 2010) segmented with the Freesurfer software (https://surfer.nmr.mgh.harvard.edu, version 7.4.1). Subcortical regions were labelled using Freesurfer’s automatic subcortical segmentation tool according to the ASEG atlas (Fischl et al., 2002). The resulting atlases were registered to diffusion space with the bbregister tool (Greve and Fischl, 2009), and ROI masks were extracted in native diffusion space.

#### 2.4.2 Edges

Edges between nodes were defined as fibre tracts connecting the ROIs, traced using probabilistic tractography in FSL (version 6.0.0). Fibre orientation estimation with the Bayesian Estimation of Diffusion Parameters Obtained by Sampling Techniques for crossing Fibres (bedpostX) tool in FSL was conducted prior to this analysis and documented elsewhere (e.g., Wendt et al., 2024). We estimated fibre tracts between ROIs with the FSL-based Probabilistic Tractography for Crossing Fibres (probtrackX2) tool (Behrens et al., 2007, Behrens et al., 2003) in network mode and native diffusion space, with stop masks defined as ventricular cerebrospinal fluid. Tracking was performed based on default probtrackX2 settings (5000 samples, 2000 steps per sample with 0.5 mm step length and curvature threshold of 0.2). The number of streamlines per tract was used as a weight measure for creating the connectivity matrix.

#### 2.4.3 Graph metrics

Graph analyses were implemented in the R Software for Statistical Computing (version 4.2.0, R Core Team, 2022) using the brainGraph package, based on igraph (Csárdi et al. 2024, Watson, 2020). Connectivity matrices with raw streamline counts were obtained from FSL. We normalised edge weights by dividing the raw streamline count by tract length (Roberts et al., 2017). Given the undirected nature of the connections, the connectivity matrix was symmetrised by averaging the upper and lower triangles. To reduce the false-positive rate in connectivity weights, we applied proportional thresholding (Bullmore and Bassett, 2011, Rubinov et al., 2010) across all individual networks, ranging from 5% to 50% density (Bullmore and Bassett, 2011) in 5% increments. This approach yielded ten connectomes per participant for network comparisons.

**Fig. 1:**
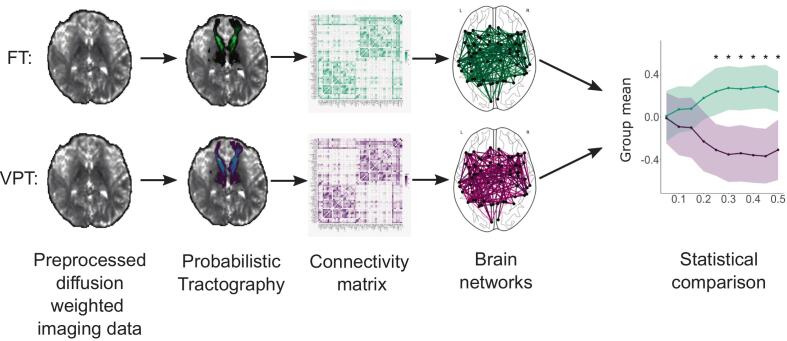
Processing pipeline for obtaining the graph metrics. *Notes*. Schematic representation of the analysis pipeline comparing network segregation and integration measures. Connectivity matrices and brain networks are averages per group; FT: full term group, VPT: very preterm group.

Three global network parameters that were shown previously to have good to excellent reliability over time (Welton et al., 2015) were calculated for each density level. Structural network segregation was measured using the weighted average clustering coefficient, which captures the network's tendency to organise into distinct clusters. The average clustering coefficient is calculated as the fraction of a node's neighbours that are also connected to each other, averaged across all nodes in the network (Watts and Strogatz, 1998). Structural network integration was assessed using two metrics. First, the unweighted average degree of the network was calculated as a basic measure of structural connectivity. This metric represents the average number of direct (binary) connections per node. The second metric, which provides additional insight into network integration, was the average weighted global efficiency of the network. Defined as the mean of the inverse shortest path lengths between all node pairs (Rubinov et al., 2010), this measure reflects the network’s potential for efficient information transfer (Drakesmith et al., 2015). In a secondary preregistered analysis, we also compared the weighted betweenness centrality of each node between groups as a measure of nodal importance. Betweenness centrality is defined as the fraction of shortest paths between any two nodes in the network that pass through the node of interest. This analysis is exploratory, as comparing 66 nodes between groups with our sample size is likely to be underpowered. Due to the scale differences in brain metrics, all brain metrics were *z*-standardised. The processing pipeline with the average connectivity matrix and brain networks per group is shown in Fig. 1.

### 2.5 Statistical analysis

Statistical analyses were also implemented in R. Group differences were analysed using an unpaired, between-subjects design. We excluded individuals with more than 50% missing values for either brain or behavioural measures from the entire analysis. Potential outliers were identified through visual inspection of bivariate associations in scatterplots. In cases where observations showed a leverage effect, we excluded observations that were > 3 standard deviations above or below the mean for either the behavioural variable or the brain metric.

#### 2.5.1 Comparison of latent cognitive control ability between the groups

The first hypothesis, testing group differences in cognitive control, was examined using a confirmatory factor analysis (CFA), implemented with the lavaan package (Rosseel, 2012) in R. We estimated cognitive control as a latent variable indicated by several behavioural task performance indicators, expert observations, and questionnaires, as listed in Section 2.2. A latent variable in CFA captures shared variance among indicators, providing insight into their relationship with the latent factor. Missing cases were handled using full information maximum likelihood estimation. To account for the skewed distributions of some indicators as confirmed by the Shapiro-Wilk’s test (for details, see Section 3), the Maximum Likelihood Robust (MLR) estimator was used for model estimation. Latent variables were identified by standardisation.

We applied standard inference criteria for structural equation modelling (SEM; Kline, 2011). Misfit between the model implied and the observed covariance matrices was firstly assessed with the χ2-value. Higher χ2*-*values indicate greater misfit between the two matrices; therefore, non-significant values or χ2 /degrees of freedom < 2.5 indicate satisfactory model fit. Additional fit indices were also considered: model misspecification per degrees of freedom was assessed with the Root Mean Square Error of Approximation (RMSEA), and the Standardised Root Mean-square Residual (SRMR) measured the average standardised deviation of model-implied versus observed covariances. We conclude a satisfactory fit with RMSEA ≤ 0.08 and SRMR ≤ 0.08. The Comparative Fit Index (CFI), indicating model improvement over a null model that assumes no correlation among indicators, was considered satisfactory at CFI ≥ 0.90 (Hooper et al., 2008).

To establish a latent cognitive control model with the indicators at hand, we first estimated a measurement model for the combined VPT and FT sample. This a priori analysis, conducted prior to preregistration, served to confirm that the construct of interest could be estimated by a robust measurement model given the available indicators and sample. No group differences were examined in this a priori analysis, and thus no hypotheses were tested prior to preregistration.

To examine group mean differences in the latent factors, and thus address the first hypothesis, we performed a multi-group CFA based on the a priori established measurement model. Latent mean structures were compared after establishing measurement invariance, and if at least partial strong invariance was achieved (Hirschfeld et al., 2014), the model was compared to one with latent variables’ means across groups constrained to equality. In interpreting the model, FT latent means were set at 0, and VPT estimates reflected differences relative to FT. Since latent variables were standardised, fully standardised regression weights indicate differences in standard deviation units compared to the reference group − here the group of FT individuals.

#### 2.5.2 Comparison of network topology between the groups

To test the second hypothesis, postulating higher segregation (average clustering coefficient) and lower integration (nodal degree and global efficiency) in the VPT group, we compared the three global network metrics per participant across density values (5–50%) between groups using two-tailed *t*-tests. We corrected α-values for false discovery rate (FDR) using the Benjamini and Hochberg (1995) procedure, as implemented in the stats package in R, to account for multiple density thresholds and metrics (e.g., Batalle et al., 2017). Effect sizes for comparing two population means were calculated with Cohen’s *d*.

We examined the betweenness centrality of nodes between groups at a fixed density level of 50%. Two-tailed *t*-test α-values were FDR-corrected for the 66 comparisons.

#### 2.5.3 Brain-behaviour associations

To address the third hypothesis, we applied a SEM approach to test associations between brain network topology metrics and latent factors of cognitive control, using the lavaan package in R. To analyse brain-behaviour associations within each group, we conducted a series of multi-group models (one for each topology feature, given anticipated multicollinearity among brain metrics). Regression weights of topology features that survived FDR-correction in their predictive power on cognitive control across the entire sample were then constrained to equivalence to test group differences. A regression analysis result was considered supportive of our hypothesis if the FDR-corrected *p*-values for the regression weights of network characteristics on latent cognitive control were ≤ 0.05 for any of the three network characteristics and two latent factors. Additionally, the regression coefficient had to exceed a minimum effect size threshold (*r* > 0.1) to be considered meaningful.

To reduce the number of models and comparisons, we aggregated results across density thresholds for the brain-behaviour analysis, yielding one value per graph metric (one for each degree, global efficiency, and clustering coefficient). In line with the preregistration, for graph metrics with robust group differences, we calculated an average across all densities for further analysis.

In a non-preregistered exploratory analysis, we re-examined the data using a subnetwork of subcortical structures within the cognitive control network—specifically, the caudate, putamen, thalamus, and cerebellum—regions that are known to be impacted by PT birth on a long-term (Kelly et al., 2023). Standardised brain metrics were averaged across density levels of 5–50% and FDR-corrected.

#### 2.5.4 Covariates

If significant group differences were identified in cognitive control, network topology, or brain-behaviour associations, we assessed whether these differences could be attributed to confounding variables: sex, family SES, optimality scores, head circumference. To control for the influence of covariates, we regressed latent factors and brain metrics in the above explained models on the covariates.

During the within team discussion phase of our findings, we evaluated the potential for further post-preregistration exploratory analyses. Hence, we examined the relationships between latent cognitive control ability and additional covariates, including ventilation at birth and INTI scores, as well as motor impairments measured at ages 6 and 8 years. Consequently, we extended our analyses by regressing the latent cognitive control factors on these four additional variables using separate SEMs, analogous to our brain-behaviour analysis. Furthermore, we performed regression analyses of brain metrics on ventilation at birth (utilizing a dummy regression to accommodate the binary nature of this variable) and INTI scores to assess their associations within the VPT cohort.

### 2.6 Deviations from the preregistration

The study was preregistered (osf.io/k4xwa). According to the preregistration, to test group differences in latent cognitive control, we estimated a model with configural measurement invariance between the groups with the factor loadings of the FT group restricted to the factor loadings of the entire sample and the VPT factor loadings estimated freely. Unfortunately, the model fit of this model was not sufficient to retain the restricted model (χ^2^(31) = 48.758, *p* = 0.022, CFI = 0.906, RMSEA = 0.090, SRMR = 0.096). We, therefore, used the model with partial strong measurement invariance between the groups without further restrictions for the final analysis of group differences.

As detailed in 2.5.3, 2.5.4, as part of the post-preregistration exploratory analyses, we added two analysis steps: First, the brain-behaviour associations were repeated within a subcortical subnetwork of the cognitive control network. Second, four additional covariates—ventilation disorder at birth, INTI score, and motor impairments at ages 6 and 8 years—were incorporated into the analysis pipeline. Sex was added as an additional covariate following feedback received during peer review. Also, the harmonisation step of the neuroimaging data across different scanners was not preregistered and is described in more detail in 2.3.1.

## 3 Results

We intended to include 146 participants with neuroimaging and behavioural data for the analysis. However, four participants were excluded due to > 50% missing behavioural data. Additionally, one participant was removed due to failure in surface reconstruction with Freesurfer, and another was excluded for meeting both of these criteria. This resulted in a final sample of 140 participants (79 FT and 61 VPT). Demographic data for the sample are provided in Table 1. To identify potential outliers, we visually inspected bivariate associations between indicators of cognitive control and graph metrics with scatter plots. No leverage effects were observed, so all data were retained for analysis. As anticipated, all indicators of cognitive control, except for ‘*bar*’ (*p* = 0.24) were not normally distributed (all *ps* < 0.02).

### 3.1 Cognitive control

In an a prior analysis to preregistration, a measurement model of cognitive control with two correlated latent factors was estimated on the entire sample, showing satisfactory model fit (χ^2^(14) = 27.765, *p* = 0.015; CFI = 0.940, RMSEA = 0.084, SRMR = 0.058). The latent variables were standardised. The first latent variable, termed ‘*speed of cognitive control’* reflected mental speed (response times) in tasks requiring mental inhibition, with moderate (λ_word_ = 0.471, λ_congruency_ = 0.379, and λ_vsat_ = 0.459) to high factor loadings (λ_interference_ = 0.902, λ_bar_ = 0.836). The second latent factor, ‘*sustained attention’* was indicated by two attention ratings (λ_trap_ = 0.715, λ_yabcl_ = 0.221) over a prolonged period of time. The two factors were significantly correlated (*r* = 0.564, *p* = 0.003). Given the identification status of the *sustained attention* factor with two indicators only, the loadings were restricted to equality.

This model was used to estimate group differences. Partial strong measurement invariance was established by releasing the congruency indicator from the equality constraint. This model did not differ significantly from the weak invariance model (Δχ^2^ = 8.404, *p* = 0.078), and most fit indices met the expected thresholds (χ^2^(36) = 53.466, *p* = 0.031; CFI = 0.908, RMSEA = 0.083, SRMR = 0.091). Despite minor deviations in RMSEA and SRMR from predefined fit criteria, this model was retained for further analysis. Additional details on measurement invariance are provided in the Supplement S3.

**Fig. 2:**
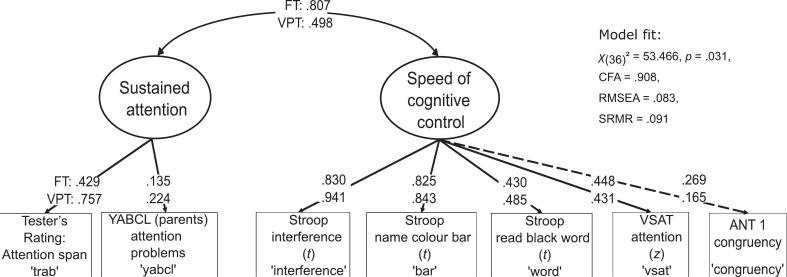
Multi-group measurement model of cognitive control. *Notes*. Schematic representation of the measurement model of cognitive control with partial strong invariance between the groups and standardised latent variables; dashed lines: indicator freed from equality constraint across groups; FT: full term group, VPT: very preterm group; YABCL: Young Adult Behaviour Check List.

We then tested mean differences between the groups, finding significant differences between the partial strong invariance model and a model with equal means (Δχ^2^ = 19.218, *p* < 0.001). This indicated significant group differences in the mean estimates of the latent variables *speed of cognitive control* and *sustained attention*. Specifically, the VPT group scored 0.742 standard deviations lower on the latent *speed of cognitive control* factor than the FT group (*p* = 0.001). The group mean differences for *sustained attention* were not significant in the measurement model (ß = −1.224, *p* = 0.334). This result may be related to the non-significant variance of the latent factor (*p* = 0.617), which suggests limited variability in this latent variable. However, mean differences at the indicator level were significant, even after FDR-correction (see Supplement Table S4.1 for details). Thus, our hypothesis that the VPT group would show lower performance on *speed of cognitive control* and *sustained attention* – as shown by indicator-level differences – was supported. The final multi-group model with partial strong measurement invariance is visualised in Fig. 2.

### 3.2 Network topology

**Fig. 3:**
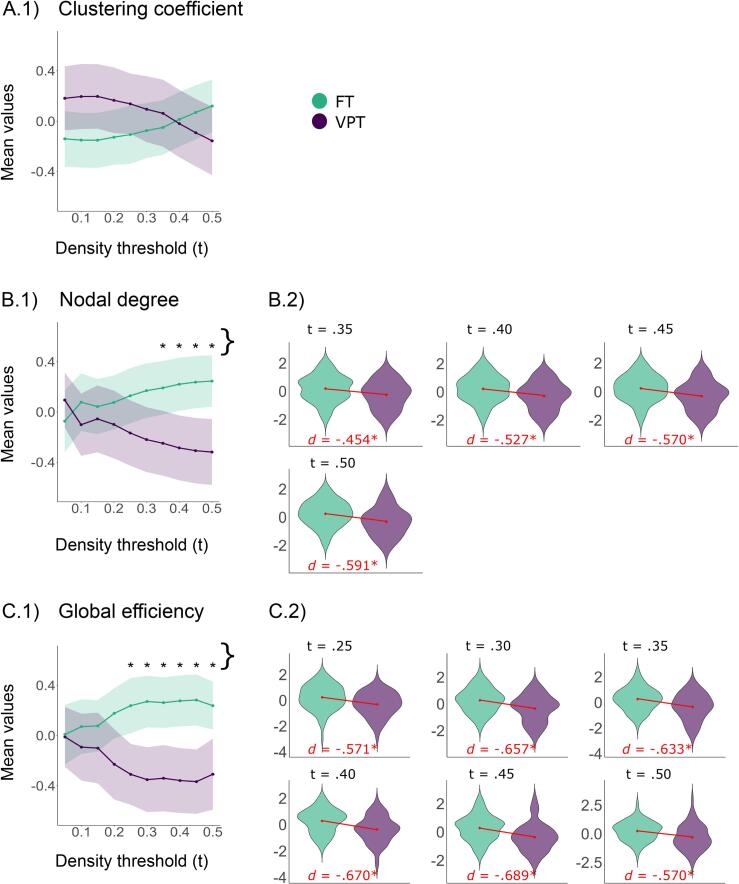
Group differences in topology metrics of the structural cognitive control brain network. *Notes*. Group differences in network segregation (A) and integration (B & C); Figures 0.1) represent mean values of the brain metrics per group (standardised) over all density values; shaded areas represent 95% confidence intervals; Note that the almost mirrored structure of the group differences arises from the z-standardisation of the variables, which is based on pooled means and standard deviations. This standardisation forces the overall (weighted) mean to zero, resulting in group means that deviate from the pooled mean in opposite directions and with similar magnitudes. Figures 0.2) show the distributions for density thresholds with significant group differences; t: density threshold, *d*: effect size Cohen’s *d*, VPT: very preterm group, FT: full term group; *: *p* ≤ 0.05 after correction for false-discovery rate.

To compare network topology in cognitive control brain networks between groups, we assessed network integration using average global efficiency and nodal degree, and network segregation using the average clustering coefficient. These metrics were compared across 10 density levels (5 – 50%). Group comparisons and distributions of brain metrics at density levels with significant group differences are shown in Fig. 3. Mean values per group, *t*-statistics, effect sizes, and (FDR-corrected) *p*-values for all comparisons are provided in Supplement Table S4.2.There were no differences in the average clustering coefficient between the groups at any density level. However, the VPT group showed a lower average degree at density levels of 35 – 50% (*p_FDR_35%_* = 0.030, *p_FDR_40%_* = 0.010, *p_FDR_45%_* = 0.004, *p_FDR_50%_* = 0.004), and lower average global efficiency at density levels of 25% – 50% (*p_FDR_25-50%_* ≤ 0.007). The trend toward lower network integration in the VPT group was also present in the harmonised subsets, although it did not reach statistical significance. Consistent with the findings in the full non-harmonised dataset, the FT group exhibited higher average degree and global efficiency than the VPT group at higher network densities (see S1.1 for the distributions of average metrics across densities within each subsample).

Thus, our hypothesis that the VPT group would exhibit lower integration and higher segregation than the FT group was supported for network integration with small to moderate effect sizes, but not for network segregation.

**Fig. 4:**
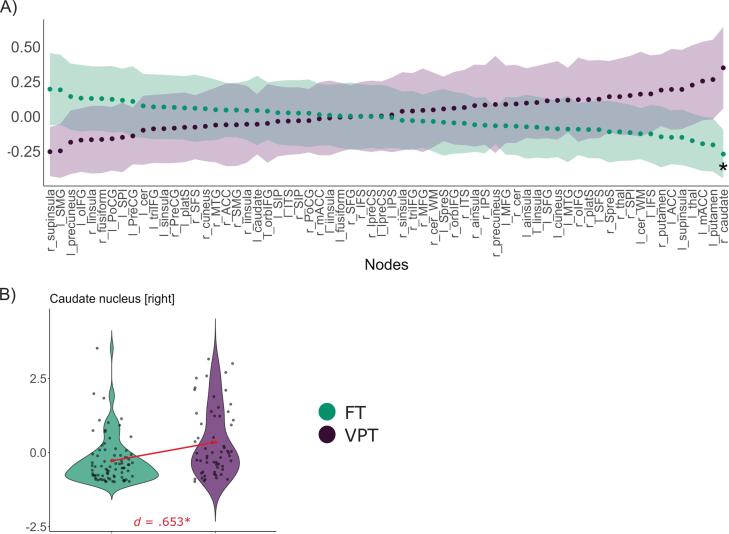
Group differences in betweenness centrality of network nodes. *Notes*. A): all nodes included in the cognitive control network at network density threshold = 50%, sorted according to the effect size of the group difference; shaded areas represent 95% confidence intervals; B): distribution of centrality measures for nodes with significant group differences; VPT: very preterm group, FT: full term group, d: effect size Cohen’s *d*; *: *p* ≤ 0.05 after correction for false-discovery rate.

In an exploratory analysis, we compared the betweenness centrality of the nodes between the groups at a density level of 50%. After FDR-correction, we found higher node centrality of the right caudate nucleus (*M_VPT_* = 0.349, *M_FT_* = -0.270, *t* = 3.63, *p_FDR_* < 0.001) in the VPT group with moderate effect sizes. Group differences in betweenness centrality of the nodes and the distribution of betweenness centrality of the node with significant group differences are illustrated in Fig. 4.

This effect remained partially robust across harmonised subsamples: higher betweenness centrality in the right caudate nucleus was consistently observed in the VPT group across all subsamples (Cohen’s *d*s = 0.402 – 0.728), although none of the node centrality differences reached statistical significance (see S1.2. for betweenness centrality over nodes for all subsamples).

### 3.3 Brain behaviour associations

**Fig. 5:**
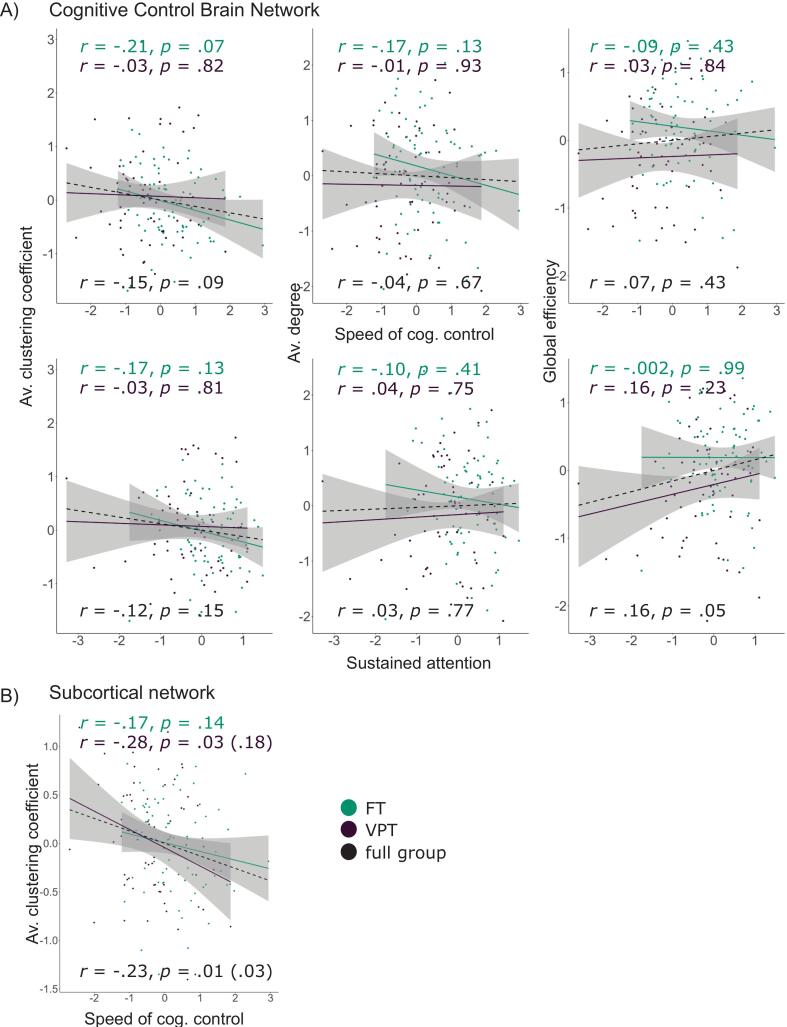
Associations between network topology and cognitive control factor scores. *Notes*. A) bivariate associations between brain metrics of the cognitive control network and factor scores of cognitive control; clustering coefficient calculated based on a density level of 20%, average degree and global efficiency calculated based on an average metric across all density levels; B) significant bivariate association between the clustering coefficient of a subcortical network known to be affected long term after preterm birth and factor scores of behavioural cognitive control; *p*-values in brackets represent *p*-values after correction for false-discovery rate; clustering coefficient calculated based on an average metric across all density levels; dashed lines: bivariate association for the full sample (VPT + FT); all brain variables are standardised; grey shaded areas represent confidence intervals; VPT: very preterm group, FT: full term group.

Next, we examined brain-behaviour associations between the latent *speed of cognitive control* and *sustained attention* factors and measures of network integration and segregation. Brain metrics were averaged across all density levels to generate a single representative value for each participant. We first investigated brain-behaviour associations in the whole sample. To reduce the number of model parameters and increase power, we constrained factor loadings to those estimated a priori to establish the measurement model, achieving satisfactory fit for all three models (brain behaviour associations for average clustering coefficient, average degree and global efficiency; χ^2^(25) = 30.418 – 32.960, *p* = 0.132 – 0.209; CFI = 0.966 – 0.976; RMSEA = 0.039 – 0.048; SRMR = 0.055 – 0.059). Regression coefficients were neither significant for the whole group nor in the multi-group model (see Supplement S5 for details), indicating that this study cannot provide evidence for brain-behaviour associations. Our hypothesis according to which latent cognitive control and network topology are associated was therefore not supported. To visualise bivariate associations between brain metrics and latent *sustained attention*/*speed of cognitive control*, we computed factor scores based on the measurement model of cognitive control (excluding brain metrics), with freely estimated factor loadings. Fig. 5A shows the brain metrics’ regression onto those factor scores for the whole group and the VPT/FT subgroups separately.

In the harmonised subsamples, a positive association between average global efficiency and latent *sustained attention* − borderline significant in the total non-harmonised sample (*r* = 0.16, *p* = 0.052) − consistently reached significance across subsamples (*rs* = 0.24-0.34, *ps* = 0.002-0.027), although the effect remained significant after correction for false discovery rate only in two out of five subsamples. Aside from a single significant positive association between average degree and latent *sustained attention* in subsample 5 (*r* = 0.23, *p* = 0.033, *p_FDR_* = 0.1), no other associations were significant (see S1.3 for bivariate brain-behaviour associations by subsample).

In the analysis of associations between latent cognitive control and brain metrics derived from a subcortical subnetwork of the cognitive control network a significant negative relationship was observed between the clustering coefficient of this subnetwork and latent *speed of cognitive control* (ß = −0.237, standard error (*SE)* = 0.163, *p* = 0.005, *p_FDR_* = 0.030; see Supplement S6 for details). The association was primarily driven by the VPT group (ß = −0.298, *SE* = 0.254, *p* = 0.030, *p_FDR_* = 0.180). Bivariate associations between the clustering coefficient of the subnetwork and *speed of cognitive control* factor scores, for the whole group and the two subgroups, are illustrated in Fig. 5B.

### 3.4 Covariates

To address potential confounding factors in the observed group differences in cognitive control and network topology, we investigated associations with sex, family SES, optimality scores, head circumference, ventilation at birth, and INTI score, with the last two assessed only in the VPT group. To control for their influence, we regressed the latent factors and brain metrics onto each covariate in separate models. In a multi-group model with partial strong invariance, regression of the *speed of cognitive control* and *sustained attention* factors on the covariates revealed no significant associations for most covariates, except for the INTI score, which showed a significant negative association with *speed of cognitive control*, albeit with a small effect size (ß = −0.258, *SE* = 0.031, *p* = 0.015, *p_FDR_* = 0.04). Hence, we can conclude that most covariates are not relevant in explaining individual differences in latent cognitive control, either for the whole group or within the separate groups. However, the intensity of neonatal treatment, as reflected by the INTI score within the VPT group, appears to have a measurable but small association with later cognitive control, specifically its speed component.

For the analyses of the neural variables, separate regression models were applied for each density threshold and brain metric, with graph metrics regressed on the covariates. None of the main or interaction effects of the covariates reached statistical significance after FDR-correction (all *p_FDR_* > 0.05), suggesting that the covariates do not contribute meaningfully to explaining individual differences in network topology and can therefore be disregarded in the interpretation.

We further examined the association between motor impairment in 6 and 8-year old participants and adult latent cognitive control. A significant negative association with small effect sizes was observed between motor impairment and *speed of cognitive control* at both 6 years (ß = −0.355, *SE* = 0.044, *p* < 0.001, *p_FDR_* < 0.001) and 8 years (ß = −0.241, *SE* = 0.035, *p* = 0.001, *p_FDR_* = 0.004) for the entire group. No significant association was found with *sustained attention*. In a multi-group SEM, the association between motor impairment at 6 years and *speed of cognitive control* was confirmed for the FT group (ß = −0.322, *SE* = 0.072, *p* = 0.005, *p_FDR_* = 0.040), but not the VPT group (ß = −0.264, *SE* = 0.057, *p* = 0.070). Notably, the association was not significant for the 8 year olds. Given 11 and 10 missing observations at 6 and 8 years, respectively in the VPT group, compared to 0 and 4 missing observations in the FT group, we repeated the analysis after imputing missing data points per group with the mice package (van Buuren and Groothuis-Oudshoorn, 2011) in R. This analysis yielded similar results. Detailed model fit indices and regression coefficients for SEMs can be found in Supplement S7.

## 4 Discussion

In this study, we examined the long-term impacts of VPT birth on cognitive control and its neural underpinnings. By employing a latent variable modelling approach to assess cognitive control ability, we aimed to minimise measurement specificity and eliminate measurement error, offering a more precise evaluation of cognitive control than in previous studies. Additionally, we explored differences in brain network characteristics between groups using graph-theoretic analysis, providing insights into potential long-term structural network alterations associated with VPT birth. Finally, we assessed brain-behaviour associations, integrating neural and behavioural data to capture their associations. We found diminished cognitive control ability and reduced network integration (global efficiency and nodal degree) in the VPT group, but no differences in network segregation (average clustering coefficient). Further, we did not find associations between network topology and cognitive control ability.

### 4.1 Group (VPT vs. FT) differences in cognitive control

As hypothesised, we found lower levels of latent cognitive control in VPT adults, hence we conclude that impairments observed until adolescence (Burnett et al., 2013) remain evident in adulthood. While some studies have suggested that lower performance on cognitive control tasks results from a developmental delay that resolves by adolescence (Aarnoudse-Moens et al., 2012, Réveillon et al., 2018), our findings, alongside evidence from other research (Nosarti et al., 2007, Wolke et al., 2019) point to a different conclusion. Specifically, we find no indication that cognitive control impairments evident in childhood vanish over adult age. This persistence is particularly concerning given recent findings suggesting no difference (Burstein et al., 2024) or even an increase in executive function deficits in more recent cohorts of PT-born individuals (Burnett et al., 2018).

Impairments in the latent *speed of cognitive control* factor were particularly pronounced, with the VPT group scoring 0.742 standard deviations lower than their FT peers. This difference exceeds the group differences of approximately 0.5 standard deviations reported in previous studies (Wolke et al., 2019), suggesting that reducing measurement biases and error amplifies the observed disparities between groups. The *speed of cognitive control* factor captures maximal performance within the context of response inhibition/interference control. It includes indicators measured through response times or with time restrictions. A common challenge in assessing mental speed is distinguishing it from motor skills, which are often impaired in PT-born individuals (Evensen et al., 2020). For example, in a go/no-go task administered to children born VPT, motor skills mediated the relationship between birth status and impaired behavioural performance (Ludyga et al., 2021). To address this potential mediation effect, we examined the impact of childhood motor impairments (assessed at 6 and 8 years) on latent cognitive control. As anticipated, motor impairments were significantly associated with the latent *speed of cognitive control* but showed no relationship with latent *sustained attention*. These effects were observed in the full sample, but were more pronounced in the FT group compared to their VPT peers, albeit with small effect sizes in all models. In line with research on deficits in motor-independent visual attention in VPT-born adults (Finke et al., 2015, Menegaux et al., 2017), these results suggest that motor impairments, at most, only partially account for the group differences in latent *speed of cognitive control*.

While the *speed of cognitive control* factor primarily reflects state-level cognitive control, focusing on response inhibition and interference resolution, the *sustained attention* factor represents typical attention performance, potentially linked to trait-like characteristics. Although *the sustained attention* factor was less elaborately measured (only two indicators) and therefore more challenging to interpret, lower performance on its indicators was found together with an increased prevalence of ADHD diagnoses in an extended adult sample from the BLS study (Breeman et al., 2016). In summary, our findings confirm that VPT birth significantly increases the likelihood of long-term challenges in cognitive control, evident at the latent construct level and even within subgroups of VPT adults who may demonstrate better adaptive capacities, as indicated by their eligibility for MRI-based studies. These differences might be rooted in alterations in brain network topology.

### 4.2 Group (VPT vs. FT) differences in network topology

Consistent with our hypothesis, we observed lower network integration in VPT compared to FT young adults. This pattern aligns with previous findings across infancy, childhood, and post-adolescence (de Almeida et al., 2021, Fischi-Gomez et al., 2016, Irzan et al., 2021, de Kieviet et al., 2021). Behavioural implications of structural network topology metrics can be inferred based on previous studies. In the healthy brain, structural integration increases during adolescence and early adulthood (Chen et al., 2013, Hagmann et al., 2010, Luna et al., 2015). These developmental changes parallel improvements in cognitive control behaviours, which continue into early to mid-adulthood (Erb et al., 2023). It has been proposed that increasing integration between specialised networks drives these cognitive control improvements over time (Luna et al., 2015). Evidence from functional networks supports this notion, suggesting that enhanced integration of the salience network in adulthood contributes to faster response times during inhibitory control tasks (Marek et al., 2015). Thus, lower network integration in the VPT group may contribute to their heightened vulnerability to cognitive control impairments, although this relationship was not directly confirmed in our study.

This reduction in global network integration likely reflects a diminished focus on long-range association fibres. This is also supported by the generally larger effect sizes at higher density levels in our study, which suggest that stronger connections within the core network in the VPT group are less affected compared to weaker long-range connections. These long-range fibres, which are essential for facilitating efficient communication across distributed brain regions, undergo critical stages of development between 20 and 40 weeks of gestation—the period during which PT birth typically occurs (Batalle et al., 2017). Long association fibres require especially extensive myelination, a process that is particularly vulnerable to disruption following PT birth (Volpe, 2009). Hence, early impairments in cellular function may interfere with the development of long-range fibres, thereby contributing to the observed reductions in network integration in PT individuals.

Long-range association fibres also play a critical role in achieving an optimal balance between minimising energy costs while maintaining anatomical integration in structural brain networks (Bullmore et al., 2012). Cui et al. (2020), explored the developmental mechanisms behind the optimisation of this trade-off, focusing on the maturation of structural brain networks—especially the cognitive control network—during adolescence and early adulthood. They demonstrated that these networks evolve to minimise theoretical energy costs associated with transitions between baseline and active states, with individuals exhibiting higher cognitive control requiring less energy for such transitions. This structural optimisation is mirrored in functional brain networks involved in cognitive control, which also exhibit lower energy demands during state transitions compared to networks supporting basic sensory functions (Saberi et al., 2024). These findings suggest that both structural and functional networks supporting cognitive control are efficiently tuned for task-related demands. The underlying processes are shaped by topological properties of the network’s architecture (Cui et al., 2020), emphasising the importance of balancing segregation and integration for optimal functioning. Thus, energy-efficient network dynamics are closely tied to structural topology, and this efficiency is crucial for supporting cognitive control. Our findings suggest that VPT individuals may be vulnerable to difficulties in optimising network integration, potentially leading to altered energy efficiency and consequent impairments in cognitive control.

Contrary to our hypothesis, we did not find group differences in network segregation, suggesting a comparable reliance on short-range connections in the VPT and FT groups. This aligns with findings by Fischi-Gomez et al. (2016), who reported no significant group differences in clustering coefficient between extremely PT and FT school-aged children. These results imply that group differences observed in earlier developmental stages may either attenuate or become less consistent with age, compared to differences in network integration. This is particularly interesting given that cognitive functions requiring distributed processing, such as cognitive control, rely more heavily on high global efficiency than on high clustering (Bullmore et al., 2012). In this context, our findings that the VPT group exhibited alterations in cognitive control and network integration, but not in network segregation, align with the broader understanding of these network properties and their functional significance.

In an exploratory analysis, we additionally examined differences in node centrality between FT and VPT individuals. Our results showed a higher centrality of the right caudate nucleus in the VPT group. This finding should be interpreted with caution, given its partial robustness after harmonisation across scanners and its contrast with previous studies that reported reduced betweenness centrality (Irzan et al., 2021) and volume (Kelly et al., 2023, Nosarti et al., 2014) in PT-born young adults. The underlying mechanisms driving structural changes in the caudate nucleus after PT birth remain incompletely understood. However, our study adds to the growing evidence that this structure, known for its critical role in executive functioning (Grahn et al., 2008), is significantly affected by premature birth in the long term.

While our study delivers novel and valuable insights into the long-term effects of VPT birth on the structural connectome, its cross-sectional design limits our ability to draw conclusions about developmental trajectories. Existing studies on the healthy brain focus on developmental processes in the brain until early adulthood. While they potentially help to understand the topological and behavioural differences between VPT- and FT-born adults observed in our study, we acknowledge that future research should adopt longitudinal designs that extend beyond adolescence to better capture these developmental trajectories and their behavioural correlates after PT birth.

### 4.3 No brain-behaviour associations for brain network segregation/integration and cognitive control

We did not find a relationship between network topology and latent cognitive control, which aligns with other brain-behaviour studies with small sample sizes. For instance, a study of school-aged children also reported no significant association between cognitive ability and clustering coefficient, though the sample size was limited to 20 VPT children (de Kieviet et al., 2021). Potential limitations due to sample size were already addressed in our study’s preregistration; however, some measures to reduce model complexity and enhance statistical power were not feasible due to challenges with model fit.

Identifying robust brain-behaviour associations remains a persistent challenge. While it seems reasonable to assume an association between brain structure and behaviour, any such relationship is likely subtle, and in many cases of a small effect size. Several factors may contribute to such weak effects, including the signal-to-noise ratios in neural data (Gratton et al., 2022) and the use of latent constructs. While latent constructs offer a more meaningful representation of cognitive abilities, they also introduce an additional layer of abstraction, making direct associations harder to detect. Furthermore, structural metrics and univariate analyses tend to yield smaller effect sizes compared to functional metrics and multivariate approaches (Marek et al., 2022). Although definitive conclusions cannot be drawn from this analysis, we argue that studies with low power still hold value for the scientific community by informing the design of larger studies, particularly in terms of variable selection and effect size estimation.

Further support for the notion that the absence of brain–behaviour associations in the full sample may not capture the complete picture comes from our additional exploratory analyses. Specifically, latent sustained attention was consistently positively associated with network integration (global efficiency) after correcting for scanner-related variability, even though the effect did not survive correction for multiple comparison in all subsamples. Also, we conducted an exploratory analysis to examine brain-behaviour associations within a subnetwork of the cognitive control brain network. This subnetwork consists of the caudate, putamen, thalamus, and cerebellum, since those structures are known to be strongly affected by PT birth in the long-term (Kelly et al., 2023). In this analysis, we identified a significant negative association between clustering coefficient, or the tendency of brain networks to form local “cliques” that indicates functional specialisation, and latent *speed of cognitive contro*l across the entire sample. While a significant association was also observed within the VPT group, this did not survive correction for FDR.

Although higher clustering was associated with poorer cognitive performance in our exploratory analyses, it is not inherently detrimental. Healthy brain networks typically exhibit small-world properties, characterised by a combination of high clustering and high efficiency (see, e.g., Bassett and Bullmore, 2017). The precise associations between preterm birth, network clustering, and cognitive performance are yet to be fully understood and this subnetwork may represent a promising target for future investigations of brain–behaviour associations.

Although this interpretation should be approached with caution, the higher association observed in the VPT group may reflect the phenomenon of ability dedifferentiation. Specifically, lower-performing groups of individuals, such as the VPT group, often demonstrate higher factor loadings on a general ability factor (Spearman, 1927, Tucker-Drob, 2009), reflecting a greater reliance on domain-general abilities. This phenomenon aligns with our findings, where the VPT group exhibited lower cognitive control scores paired with generally higher factor loadings. The increased variance observed in cognitive control factors within the VPT group may provide a more robust foundation for identifying biomarkers (Liu et al., 2022) leading to increased brain-behaviour associations.

### 4.4 Limitations and outlook

The findings of this study should be considered alongside several important factors that may influence interpretation. First, the observed effects may be underestimated due to the inclusion criteria for MRI, which limited the VPT sample to individuals meeting specific imaging requirements. This subsample of VPT individuals was less likely to have experienced severe impairments or multiple complications at birth and had a higher intelligence quotient than the general VPT group in the BLS sample (Menegaux et al., 2020). As a result, it may not fully represent the general VPT population. However, the potentially conservative nature of our findings suggests that analyses on a more representative sample could yield even stronger effects, underscoring the need for further research.

The relatively modest sample size should also be taken into account when interpreting its findings. Lifespan data from at-risk populations, such as individuals born preterm followed into early adulthood, are difficult to obtain, and – as already discussed above – the acquisition of neuroimaging data is associated with additional challenges. Although the sample size of the present study is relatively large compared with similar work in the field, it is still small and can be taken as a study to inform larger multi-centre trials in the future. Small samples are not only prone to miss the identification of small effects, but they are also more susceptible to the influence of outliers, which can reduce the robustness of observed effects and increase the risk of inflated effect sizes. Although the study was preregistered to mitigate the risks of selective reporting, the limited sample size nonetheless results in less precise estimates. Furthermore, these findings may be difficult to generalise to the population. A smaller cohort is less likely to capture the full spectrum of demographic and individual variability present in the wider population of preterm born individuals. Consequently, while the observed association patterns provide valuable knowledge, they should be interpreted as preliminary and used to inform future research. Future research should employ larger and more diverse samples to replicate these findings and confirm their applicability in different contexts. This note is particularly relevant at a time when it is becoming increasingly common to collect and store clinical data and population cohort data across multiple clinics.

A further limitation relates to the use of probabilistic tractography as the foundation for our graph-theoretical analysis. While it is among the most advanced and widely used approaches for reconstructing white matter pathways in vivo, it does not provide a direct one-to-one mapping of biological acons (Jeurissen et al., 2019, Jones et al., 2013). In our study, the number of reconstructed streamlines connecting two regions was used as the basis for computing graph metrics. This probabilistic estimate is influenced by multiple factors, including the volume of the connected regions, local fibre geometry, and algorithm-specific parameters (Le Bihan, 2024, Le Bihan et al., 2012, Maier-Hein et al., 2017). In addition, probabilistic tractography is susceptible to false-positive connections, whereby streamlines propagate into adjacent but anatomically distinct pathways (Jeurissen et al., 2019); an issue commonly addressed through thresholding procedures. Such false-positive connections can bias binary graph-theoretical measures of network topology, leading to systematic overestimation of node degree and global efficiency and underestimation of the clustering coefficient (Zalesky et al., 2016). Because an identical processing pipeline was applied to both groups in our analysis, these methodological biases will affect the estimates of both groups similarly. Consequently, we interpret our findings as the best approximation available in-vivo of underlying biology and as relative differences between groups rather than absolute representations of underlying anatomical connectivity. Future studies employing multi-shell diffusion data may further mitigate these limitations by adopting more advanced modelling approaches, such as multi-tissue constrained spherical deconvolution, which provide a more specific characterization of the white-matter fibre architecture (Dell'Acqua and Tournier, 2019).

Furthermore, graph theory-based analyses of network topology involve numerous methodological decisions that could influence outcomes (Bullmore and Bassett, 2011). These decisions span the entire analysis pipeline, from data acquisition to preprocessing, postprocessing, graph construction, and statistical analyses, and currently no universally accepted standards exist (see, e.g., Yeh et al., 2021or an overview of potential fitfalls). Additionally, the interpretation of graph metrics relies on several assumptions, such as the notion that information travels along the shortest paths in the brain, or that a higher number of reconstructed streamlines reflects faster or more reliable signal transmission (e.g. Zhang et al., 2022) that might not always hold true. It is important to keep these limitations in mind, when interpreting results from tractography-based graph analyses.

Despite these limitations, our study has several notable strengths that enhance the robustness and reliability of its findings. First, the dataset used is unique in its long-term, prospective follow-up of VPT individuals into adulthood, allowing for an unprecedented investigation of brain network topology in adulthood. Second, while the imaging data were acquired on older scanners, the entire dataset was re-preprocessed using state-of-the-art pipelines, substantially improving data quality compared to earlier work on this cohort. Third, to mitigate potential biases and enhance the reliability of our results, we preregistered our analytical pipeline, thus reducing arbitrariness and ensuring methodological transparency. Finally, despite their limitations, graph-theoretical metrics have proven highly valuable in the study of structural brain networks, owing to their interpretability and sensitivity to brain–behavior associations as well as neurodevelopmental disorders.

Looking ahead, longitudinal studies of brain development will be essential for understanding the trajectories of network parameters following PT birth. Such research will deepen our understanding of neural development in this population and also provide insights into how network topology evolves over the lifespan.

### 4.5 Conclusion

Despite the inherent complexity of interpreting network parameters, the present study offers valuable insights into the long-term impacts of VPT birth on brain topology and cognitive control. Our findings confirm that VPT birth substantially heightens the risk of enduring cognitive control challenges. Notably, the observed differences in topological network parameters between VPT and FT groups, along with the VPT group's diminished cognitive control performance, suggest that optimising the structural cognitive control network may pose unique challenges for individuals born VPT. Given the sparse literature on network topology in PT-born adults, this study takes a significant step toward unravelling the neural development processes associated with VPT birth.

## Declaration of generative AI and AI-assisted technologies in the writing process

During the preparation of this work the authors used ChatGPT (OpenAI) and DeepL in order to improve readability and language of the manuscript. After using this tool/service, the authors reviewed and edited the content as needed and take full responsibility for the content of the published article.

## Funding sources

Dieter Wolke is supported by a UK Research and Innovation (UKRI) grant (EP/X023206/1) under the UK government’s Horizon Europe funding guarantee for ERC-Advanced Grants. Christian Sorg, Aurore Menegaux, and Dennis M. Hedderich report financial support was provided by the Deutsche Forschungsgemeinschaft (DFG, SO 1336/1–1 to C.S., ME 5894/2–1 to A.M., HE 8967/3–1 to D.M.H.). Peter Bartmann, Dieter Wolke, and Christian Sorg report financial support was provided by the German Federal Ministry of Education and Science (BMBF 01ER0801 to P.B. and D.W. and BMBF 01ER0803 to C.S.). Christian Sorg and Dennis M. Hedderich report financial support was provided by the Commission for Clinical Research, Technical University of Munich (KKF 8,765,162 to C.S and KKF8700000474 to D.M.H.). Jil Wendt and Merle Marek were supported by the German Academic Scholarship Foundation**.** Jil Wendt was also supported by the Else Kröner-Fresenius-Stiftung (Promotionsprogramm Translationale Medizin of the Technical University of Munich) and Merle Marek was additionally supported by the Heinz Neumüller Stiftung.

## CRediT authorship contribution statement

**Merle J. Marek:** Conceptualization, Methodology, Data curation, Formal analysis, Writing – original draft. **Dieter Wolke:** Writing – review & editing, Supervision, Funding acquisition, Data curation. **Christian Sorg:** Writing – review & editing, Funding acquisition, Data curation. **Jil Wendt:** Writing – review & editing, Formal analysis, Data curation. **Aurore Menegaux:** Writing – review & editing, Formal analysis, Data curation. **Dennis Hedderich:** Writing – review & editing, Formal analysis, Data curation. **Peter Bartmann:** Writing – review & editing, Funding acquisition, Data curation. **Micha Burkhardt:** Writing – review & editing, Formal analysis. **Andrea Hildebrandt:** Writing – review & editing, Supervision, Resources, Methodology, Conceptualization. **Axel Heep:** Writing – review & editing, Supervision, Resources, Methodology, Conceptualization.

## Declaration of competing interest

The authors declare that they have no known competing financial interests or personal relationships that could have appeared to influence the work reported in this paper.

## Data Availability

The dataset used in this study was made available to the authors by the study team of the responsible universities (University of Warwick, University Hospital Bonn, and Technical University of Munich) for the purposes of this study. Readers seeking access to the data should contact Prof. Dr. Dr. Peter Bartmann at the Department of Neonatology at the University Hospital in Bonn and Prof. Dr. Dr. Dieter Wolke at the Department of Psychology at the University of Warwick in Warwick. Access will be granted to named individuals in accordance with ethical procedures governing the reuse of clinical data, including completion of a formal data sharing agreement and approval of the local ethics committee. The study was preregistered prior to the analysis (https://osf.io/k4xwa) and analysis scripts are available (https://osf.io/2nvdp).

## Glossary

ACC: anterior cingulate cortex
ADHD: attention deficit hyperactivity disorder
ANT: Attention Network Task
BLS: Bavarian Longitudinal Study
CFA: confirmatory factor analysis
CFI: comparative fit index
DWI: diffusion weighted imaging
FDR: false discovery rate
FT: full term
GA: gestational age
INTI: intensity of neonatal treatment index
MCC: mid-cingulate cortex
MRI: magnetic resonance imaging
PFC: prefrontal cortex
PT: preterm
RMSEA: root mean square error of approximation
ROI: region of interest
SEM: structural equation model
SES: socioeconomic status
SRMR: standardised root mean-square residual
TOMI: Revised Test of Motor Impairment
TRAB: Tester’s Rating of Adult Behavior
T1w: T1 weighted
VPT: very preterm
VSAT: Visual Search and Attention Test
YABCL: Young Adult Behavior Checklist
