# Supplementary material for: Structural network topology and cognitive control in very preterm born young adults
Source: Neuroimage Clin. 2026 Jan 20;49:103952. doi: 10.1016/j.nicl.2026.103952 (PMC12860724; doi:10.1016/j.nicl.2026.103952)
Supplement: Supplementary Data 1 [file mmc1.docx]

**Supplement**

**S1 Results of harmonised datasets to reduce scanner effects on the analysis of neural data**

**Figure S1.1 Group differences in topology metrics of the structural cognitive control brain network in five balanced samples per scanner**

**
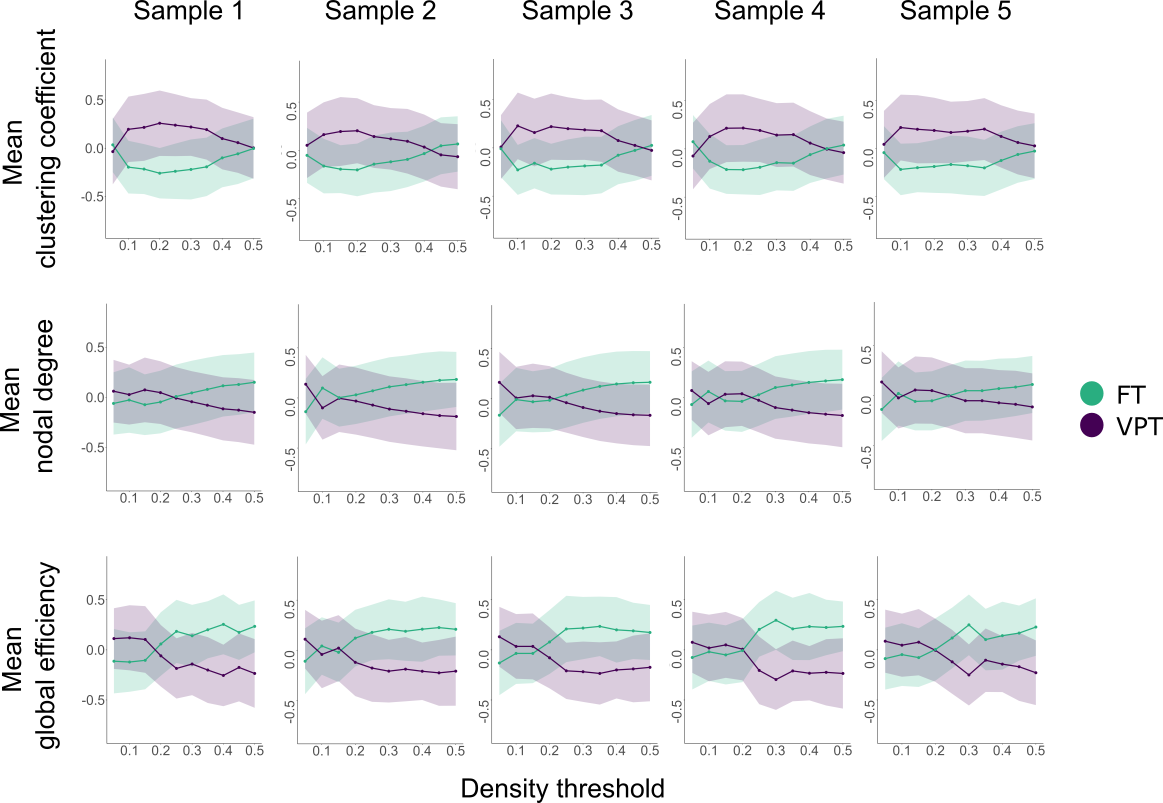
**

**Figure S1.2 Group differences in betweenness centrality of network nodes in five balanced samples per scanner**


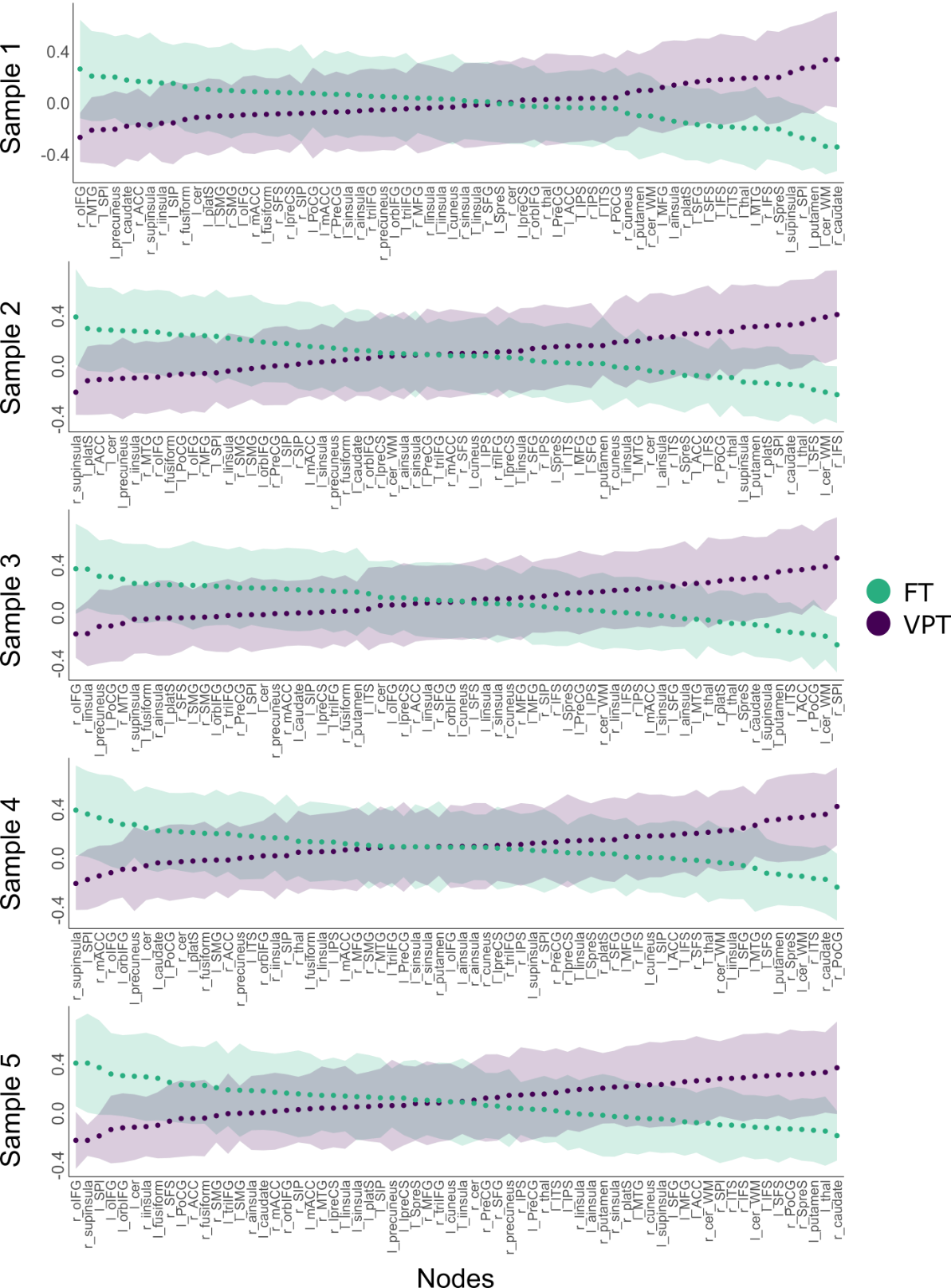


**Figure S1.3 Associations between network topology and cognitive control factor scores in five balanced samples per scanner**

**
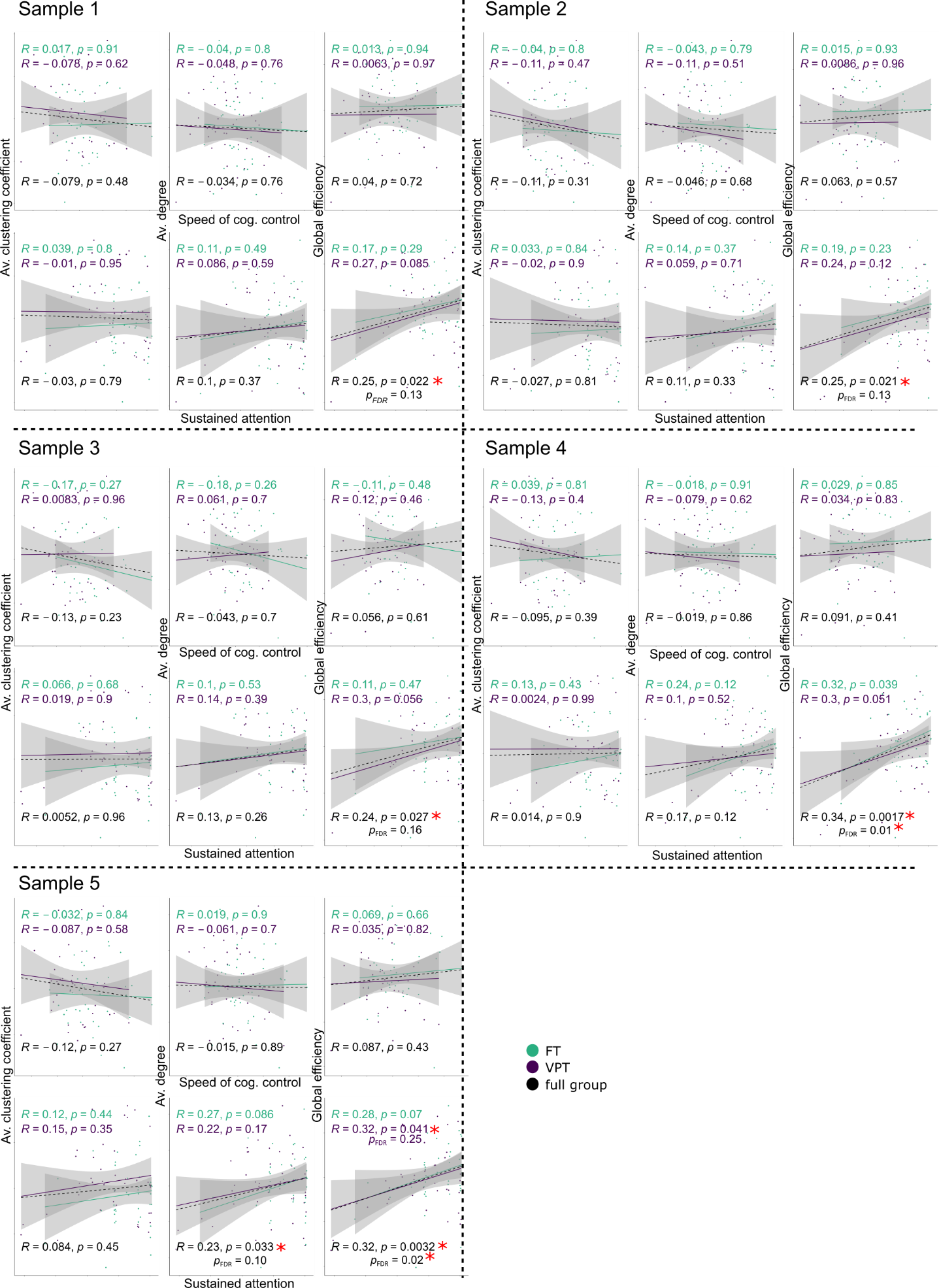
**

*Notes*. S1.2) Group differences in network segregation (clustering coefficient) and integration (nodal degree and global efficiency) for each random sample. Mean values of the brain metrics per group (standardised) are represented over all density values. S1.2) All nodes included in the cognitive control network at network density threshold = 50%, sorted according to the effect size of the group difference. S1.3) bivariate associations between brain metrics of the cognitive control network and factor scores of cognitive control. Brain metrics were calculated based on an average metric across all density levels. Dashed lines: bivariate association for the full sample (VPT + FT); Red asterisks: *p* or *p_FDR_* ≤ 0.05; all brain variables are standardised; grey shaded areas represent confidence intervals. Sampling procedure for S1.1- S1.3: Two scanners were disregarded (Bonn Achieva and Munich Ingenia) due to the low number of participants in the smaller group. For the remaining scanners (Bonn Ingenia and Munich Achieva), balanced samples were created by randomly downsampling the larger group (*N_FT_* = 42, *N_VPT_* = 42). shaded areas represent 95% confidence intervals; VPT: very preterm group, FT: full term group, *p_FDR_*: *p*-value corrected for false discovery rate.

**Table S2. Nodes of the cognitive control network**

| **Brain areas of the cognitive control network** | **Included regions (Destrieux atlas indices)** |
| --- | --- |
| **Cortical areas (bilateral)** | |
| Dorsolateral prefrontal cortex | Opercular part of the inferior frontal gyrus (12)  Orbital part of the inf. Frontal gyrus (13)  Triangular part of the inf. Frontal gyrus (14)  Middle frontal gyrus (15)  Superior frontal gyrus (16)  Precentral gyrus (29)  Inferior frontal sulcus (52)  Superior frontal sulcus (54)  Inferior precentral sulcus (68)  Superior precentral sulcus (69) |
| Insula | Long insular gyrus and central sulcus of the insula (17)  short insular gyrus (18)  Anterior segment of the circular sulcus of the insula (47)  Inferior segment of the circular sulcus of the insula (48)  Superior segment of the circular sulcus of the insula (49) |
| Anterior cingulate cortex | Anterior part of the cingulate gyrus and sulcus (6)  middle-anterior part of the cingulate gyrus and sulcus (7) |
| Parietal regions | Supramarginal gyrus (26)  Superior parietal lobule (27)  Postcentral gyrus (28)  Precuneus (30)  Posterior ramus (or segment) of the lateral sulcus (or fissure) (41)  Sulcus intermedius primus (55)  Intraparietal sulcus and transverse parietal sulci (56) |
| Infero-temporal cortex | Lateral occipito-temporal gyrus (fusiform gyrus) (21)  Middle temporal gyrus (38)  Inferior temporal sulcus (72) |
| Occipital | Cuneus (11) |
| **Subcortical areas** | |
| Basal ganglia | caudate  putamen |
| Thalamus | Thalamus |
| Cerebellum | cerebellum white matter  cerebellum cortex |
|  |  |

**S3 Measurement invariance**

To test group differences, we first established measurement invariance. The model fit for configural invariance was satisfactory (χ²(29) = 40.344, *p* = .078; CFI = .940, RMSEA = .075, SRMR = .068). The fit of the weak invariance model did not significantly differ from the configural model (∆χ² = 4.166, *p* = .244). However, the model with strong measurement invariance differed significantly from the weak invariance model (∆χ² = 17.366, *p* = .004). Consequently, we examined the indicators to identify the primary source of misfit between the model-implied and observed mean structure, which was located in the congruency indicator.

**S4 Group differences**

**Table S4.1 Indicators of Behavioural cognitive control**

| Variable | Mean VPT | Mean FT | *t*-Statistic | *p*-value | *p*-value  FDR-corrected |
| --- | --- | --- | --- | --- | --- |
| Stroop: interference | **51.16** | **55.06** | **3.28** | **0.001** | **0.002**** |
| Stroop: colour word | **50.26** | **52.54** | **2.25** | **0.026** | **0.030*** |
| Stroop: colour line | **50.92** | **56.41** | **3.66** | **0.000** | **0.000***** |
| ANT: congruency | **211.00** | **239.89** | **3.59** | **0.001** | **0.002**** |
| VSAT | **92.44** | **99.47** | **2.57** | **0.011** | **0.015*** |
| TRAB: attention | **7.88** | **8.20** | **2.18** | **0.031** | **0.031*** |
| Parent attention | **12.04** | **13.64** | **3.20** | **0.002** | **0.004**** |

*Notes*. *p*-value FDR-corrected: corrected for false discovery rate. Comparisons printed in bold show significant group differences; VPT: very preterm group, FT: full term group;

*: *p* ≤ 0.05, **: *p* ≤ 0.01, ***: *p* ≤ 0.001.

**Table S4.2 Graph metrics**

| Variable | Density | Mean VPT | Mean FT | *t*-Statistic | Cohen’s *d* | *p*-value | *p*-value  FDR-corrected |
| --- | --- | --- | --- | --- | --- | --- | --- |
| Average degree | 0.05 | 0.095 | -0.073 | 1.02 | 0.170 | 0.308 | 0.406 |
|  | 0.10 | -0.101 | 0.078 | -1.06 | -0.181 | 0.290 | 0.406 |
|  | 0.15 | -0.055 | 0.043 | -0.57 | -0.098 | 0.571 | 0.612 |
|  | 0.20 | -0.099 | 0.076 | -1.02 | -0.177 | 0.311 | 0.406 |
|  | 0.25 | -0.167 | 0.129 | -1.74 | -0.300 | 0.085 | 0.150 |
|  | 0.30 | -0.220 | 0.170 | -2.31 | -0.399 | 0.022 | 0.055 |
|  | **0.35** | **-0.249** | **0.192** | **-2.63** | **-0.454** | **0.010** | **0.030*** |
|  | **0.40** | **-0.286** | **0.221** | **-3.04** | **-0.527** | **0.003** | **0.010**** |
|  | **0.45** | **-0.308** | **0.238** | **-3.28** | **-0.570** | **0.001** | **0.004**** |
|  | **0.50** | **-0.319** | **0.246** | **-3.39** | **-0.591** | **0.001** | **0.004**** |
| Clustering coefficient | 0.05 | 0.182 | -0.141 | 1.91 | 0.328 | 0.058 | 0.116 |
|  | 0.10 | 0.196 | -0.151 | 2.05 | 0.354 | 0.042 | 0.090 |
|  | 0.15 | 0.197 | -0.152 | 2.07 | 0.356 | 0.041 | 0.090 |
|  | 0.20 | 0.166 | -0.128 | 1.73 | 0.299 | 0.085 | 0.150 |
|  | 0.25 | 0.138 | -0.107 | 1.46 | 0.247 | 0.146 | 0.231 |
|  | 0.30 | 0.095 | -0.074 | 0.98 | 0.170 | 0.327 | 0.408 |
|  | 0.35 | 0.063 | -0.049 | 0.65 | 0.112 | 0.519 | 0.577 |
|  | 0.40 | -0.019 | 0.015 | -0.20 | -0.034 | 0.844 | 0.873 |
|  | 0.45 | -0.091 | 0.070 | -0.93 | -0.162 | 0.354 | 0.408 |
|  | 0.50 | -0.157 | 0.121 | -1.61 | -0.281 | 0.110 | 0.183 |
| Global efficiency | 0.05 | -0.011 | 0.008 | -0.11 | -0.019 | 0.912 | 0.912 |
|  | 0.10 | -0.092 | 0.071 | -0.95 | -0.164 | 0.345 | 0.408 |
|  | 0.15 | -0.100 | 0.077 | -1.02 | -0.179 | 0.311 | 0.406 |
|  | 0.20 | -0.229 | 0.177 | -2.36 | -0.416 | 0.020 | 0.055 |
|  | **0.25** | **-0.309** | **0.238** | **-3.34** | **-0.571** | **0.001** | **0.004**** |
|  | **0.30** | **-0.351** | **0.271** | **-3.78** | **-0.657** | **0.000** | **0.000***** |
|  | **0.35** | **-0.340** | **0.262** | **-3.62** | **-0.633** | **0.000** | **0.000***** |
|  | **0.40** | **-0.358** | **0.276** | **-3.85** | **-0.670** | **0.000** | **0.000***** |
|  | **0.45** | **-0.367** | **0.283** | **-3.96** | **-0.689** | **0.000** | **0.000***** |
|  | **0.50** | **-0.308** | **0.238** | **-3.21** | **-0.570** | **0.002** | **0.007**** |

*Notes*. *p*-value FDR-corrected: corrected for false discovery rate for density thresholds and brain metrics. Comparisons printed in bold show significant group differences; VPT: very preterm group, FT: full term group;

*: *p* ≤ 0.05, **: *p* ≤ 0.01, ***: *p* ≤ 0.001.

**S5 Brain-behaviour associations between latent cognitive control and topology of the structural cognitive control brain network**

**Table S5.1 Whole group structural equation model (SEM)**

|  | Model fit | | | | | | Regression weights | | | | | |
| --- | --- | --- | --- | --- | --- | --- | --- | --- | --- | --- | --- | --- |
|  |  |  |  |  |  |  | Speed of cognitive control | | | Sustained attention | | |
|  | χ² | *df* | *p* | CFI | RMSEA | SRMR | ß | *p* | *se* | ß | *p* | *se* |
| Clustering coef. | 30.623 | 25 | 0.202 | 0.975 | 0.040 | 0.055 | -0.152 | 0.090 | 0.117 | -0.116 | 0.359 | 0.164 |
| Degree | 30.418 | 25 | 0.209 | 0.976 | 0.039 | 0.055 | -0.043 | 0.634 | 0.100 | 0.072 | 0.583 | 0.144 |
| Global efficiency | 32.960 | 25 | 0.132 | 0.966 | 0.048 | 0.059 | 0.061 | 0.534 | 0.133 | 0.242 | 0.067 | 0.185 |

**Table S5.2: Multigroup SEM**

|  | Model fit | | | | | | Regression weights | | | | | | | | | | | |
| --- | --- | --- | --- | --- | --- | --- | --- | --- | --- | --- | --- | --- | --- | --- | --- | --- | --- | --- |
|  |  |  |  |  |  |  | VPT | | | | | | FT | | | | | |
|  |  |  |  |  |  |  | Speed of cognitive control | | | Sustained attention | | | Speed of cognitive control | | | Sustained attention | | |
|  | χ² | *df* | *p* | CFI | RMSEA | SRMR | ß | *p* | *se* | ß | *p* | *se* | ß | *p* | *se* | ß | *p* | *se* |
| Clustering coef. | 63.785 | 45 | 0.034 | 0.901 | 0.077 | 0.087 | -0.021 | 0.885 | 0.215 | -0.032 | 0.856 | 0.153 | -0.240 | 0.069 | 0.179 | -0.252 | 0.586 | 0.633 |
| Degree | 58.641 | 45 | 0.083 | 0.926 | 0.066 | 0.084 | -0.022 | 0.882 | 0.189 | 0.076 | 0.656 | 0.109 | -0.199 | 0.120 | 0.147 | -0.046 | 0.898 | 0.401 |
| Global efficiency | 60.109 | 45 | 0.065 | 0.919 | 0.069 | 0.085 | 0.001 | 0.993 | 0.213 | 0.255 | 0.386 | 0.266 | -0.112 | 0.382 | 0.199 | 0.145 | 0.709 | 0.611 |

*Notes*. Speed of cognitive control: latent factor of cognitive control for mental speed, sustained attention: latent factor of cognitive control for sustained attention, factor loadings are constrained to those of the whole group in S4.1 and to partial strong invariance in S4.2; ß: fully standardised regression coefficients, se: standard error, VPT: very preterm group, FT: full term group; brain variables are *z*-standardised; Clustering coef. (clustering coefficient, network segregation): brain metrics retrieved from density threshold of 20%; global efficiency & degree (network integration): brain metrics calculated based on an average over all values 5-50% density thresholds.

**S6 Brain-Behaviour associations between latent cognitive control and topology of a subcortical subnetwork (exploratory analysis)**

**Table S6.1 Whole group SEM**

|  | Model fit | | | | | | Regression weights | | | | | |
| --- | --- | --- | --- | --- | --- | --- | --- | --- | --- | --- | --- | --- |
|  |  |  |  |  |  |  | Speed of cognitive control | | | Sustained attention | | |
|  | χ² | *df* | *p* | CFI | RMSEA | SRMR | ß | *p* (FDR-corrected) | *se* | ß | *p* | *se* |
| Clustering coef. | 35.028 | 25 | 0.088 | 0.958 | 0.054 | 0.058 | **-0.237** | **0.005 (0.030)** | **0.163** | -0.062 | 0.617 | 0.230 |
| Degree | 33.226 | 25 | 0.126 | 0.964 | 0.048 | 0.059 | -0.018 | 0.841 | 0.127 | 0.096 | 0.476 | 0.190 |
| Global efficiency | 34.673 | 25 | 0.094 | 0.958 | 0.053 | 0.060 | -0.024 | 0.809 | 0.265 | -0.024 | 0.862 | 0.374 |

**Table S6.2 Multigroup SEM**

|  | Model fit | | | | | | Regression weights | | | | | | | | | | | |
| --- | --- | --- | --- | --- | --- | --- | --- | --- | --- | --- | --- | --- | --- | --- | --- | --- | --- | --- |
|  |  |  |  |  |  |  | VPT | | | | | | FT | | | | | |
|  |  |  |  |  |  |  | Speed of cognitive control | | | Sustained attention | | | Speed of cognitive control | | | Sustained attention | | |
|  | χ² | *df* | *p* | CFI | RMSEA | SRMR | ß | *p* (FDR-corrected) | *se* | ß | *p* | *se* | ß | *p* | *se* | ß | *p* | *se* |
| Clustering coef. | 61.653 | 45 | 0.050 | 0.913 | 0.073 | 0.085 | **-0.298** | **0.030 (0.180)** | **0.254** | -0.041 | 0.813 | 0.153 | -0.191 | 0.126 | 0.274 | -0.092 | 0.733 | 0.583 |
| Degree | 66.166 | 45 | 0.022 | 0.889 | 0.082 | 0.088 | -0.108 | 0.412 | 0.193 | 0.046 | 0.802 | 0.145 | -0.079 | 0.515 | 0.190 | 0.122 | 0.746 | 0.589 |
| Global efficiency | 66.620 | 45 | 0.020 | 0.886 | 0.083 | 0.091 | -0.068 | 0.659 | 0.433 | 0.021 | 0.920 | 0.302 | -0.072 | 0.582 | 0.377 | -0.206 | 0.428 | 0.767 |

*Notes*. Brain metrics derived from a subcortical subnetwork including the putamen, caudate nucleus, thalamus, and cerebellum, which is known to be affected long-term by PT birth; Speed of cognitive control: latent factor of cognitive control for mental speed, sustained attention: latent factor of cognitive control for sustained attention, factor loadings are constrained to those of the whole group in S5.1 and to partial strong invariance in S5.2; ß: fully standardised regression coefficients, se: standard error, VPT: very preterm group, FT: full term group, *p* (FDR-corrected): *p*-values corrected for false-discovery rate; Brain variables are *z*-standardised; Clustering coef. (clustering coefficient, network segregation) as well as global efficiency & degree (network integration): brain metrics calculated based on an average over all values 5-50% density thresholds.

**S7 Post-preregistration exploratory analyses of associations between latent cognitive control and additional covariates**

**Table S7.1 Whole group SEM**

|  | Model fit | | | | | | Regression weights | | | | | | |
| --- | --- | --- | --- | --- | --- | --- | --- | --- | --- | --- | --- | --- | --- |
|  |  |  |  |  |  |  | Speed of cognitive control | | | Sustained attention | | |  |
|  | χ² | *df* | *p* | CFI | RMSEA | SRMR | ß | *p* (FDR-corrected) | *se* | ß | *p* | *se* |  |
| Ventilation disorder at birth | 25.366 | 25 | 0.442 | 0.996 | 0.015 | 0.076 | -0.202 | 0.095 | 0.260 | -0.129 | 0.493 | 0.398 |  |
| INTI score | 26.911 | 25 | 0.360 | 0.982 | 0.036 | 0.085 | -0.258 | **0.015 (0.040)** | 0.031 | -0.314 | 0.069 | 0.052 |  |
| Motor impairment 6 y. | 46.081 | 25 | 0.006 | 0.902 | 0.082 | 0.089 | -0.355 | **0.000 (0.000)** | 0.044 | -0.209 | 0.063 | 0.053 |  |
| Motor impairment 8 y. | 44.419 | 25 | 0.010 | 0.910 | 0.077 | 0.079 | -0.241 | **0.001 (0.004)** | 0.035 | -0.126 | 0.250 | 0.050 |  |

**Table S7.2 Multigroup SEM**

|  | Model fit | | | | | | Regression weights | | | | | | | | | | | |
| --- | --- | --- | --- | --- | --- | --- | --- | --- | --- | --- | --- | --- | --- | --- | --- | --- | --- | --- |
|  |  |  |  |  |  |  | VPT | | | | | | FT | | | | | |
|  |  |  |  |  |  |  | Speed of cognitive control | | | Sustained attention | | | Speed of cognitive control | | | Sustained attention | | |
|  | χ² | *df* | *p* | CFI | RMSEA | SRMR | ß | *p* | *se* | ß | *p* | *se* | ß | *p* (FDR-corrected) | *se* | ß | *p* | *se* |
| Motor impairment 6 y. | 69.503 | 46 | 0.014 | 0.861 | 0.09 | 0.095 | -0.264 | 0.070 | 0.057 | -0.127 | 0.418 | 0.060 | -0.322 | **0.005 (0.040)** | 0.072 | -0.144 | 0.660 | 0.081 |
| Motor impairment 8 y. | 75.708 | 46 | 0.004 | 0.846 | 0.10 | 0.095 | -0.170 | 0.141 | 0.042 | -0.042 | 0.773 | 0.052 | -0.204 | 0.051 | 0.066 | 0.001 | 0.997 | 0.056 |

*Notes*. Ventilation at birth (dichotomous variable) and INTI score are only assessed in the VPT group; Speed of cognitive control: latent factor of cognitive control for mental speed, sustained attention: latent factor of cognitive control for sustained attention, factor loadings are constrained to those of the whole group in S6.1 and to partial strong invariance in S6.2; ß: fully standardised regression coefficients, se: standard error, VPT: very preterm group, FT: full term group, *p* (FDR-corrected): *p*-values corrected for false-discovery rate.
